# Chromium carbides and cyclopropenylidenes†

**DOI:** 10.1039/d1sc04910k

**Published:** 2021-10-08

**Authors:** Takashi Kurogi, Keiichi Irifune, Kazuhiko Takai

**Affiliations:** Division of Applied Chemistry, Graduate School of Natural Science and Technology, Okayama University 3-1-1 Tsushimanaka, Kita-ku Okayama 700-8530 Japan kurogi@org.kuchem.kyoto-u.ac.jp ktakai@cc.okayama-u.ac.jp

## Abstract

Carbon tetrabromide can be reduced with CrBr_2_ in THF to form a dinuclear carbido complex, [CrBr_2_(thf)_2_)][CrBr_2_(thf)_3_](μ-C), along with formation of [CrBr_3_(thf)_3_]. An X-ray diffraction (XRD) study of the pyridine adduct displayed a dinuclear structure bridged by a carbido ligand between 5- and 6-coordinate chromium centers. The carbido complex reacted with two equivalents of aldehydes to form α,β-unsaturated ketones. Treatment of the carbido complex with alkenes resulted in a formal double-cyclopropanation of alkenes by the carbido moiety to afford spiropentanes. Isotope labeling studies using a ^13^C-enriched carbido complex, [CrBr_2_(thf)_2_)][CrBr_2_(thf)_3_](μ-^13^C), identified that the quaternary carbon in the spiropentane framework was delivered by carbide transfer from the carbido complex. Terminal and internal alkynes also reacted with the carbido complex to form cyclopropenylidene complexes. A solid-state structure of the diethylcyclopropenylidene complex, prepared from 3-hexyne, showed a mononuclear cyclopropenylidene chromium(iii) structure.

## Abstract 2

Carbon tetrabromide can be reduced with CrBr_2_ in THF to form a dinuclear carbido complex, [CrBr_2_(thf)_2_)][CrBr_2_(thf)_3_](μ-C), along with formation of [CrBr_3_(thf)_3_].

## Graphical abstract

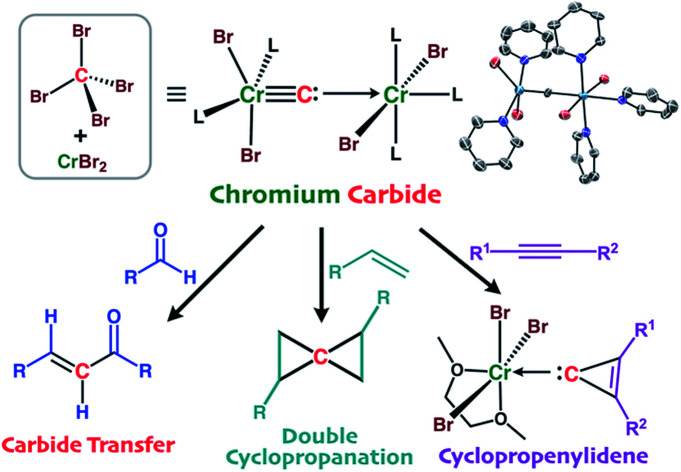

## Introduction

**Fig. 1:**
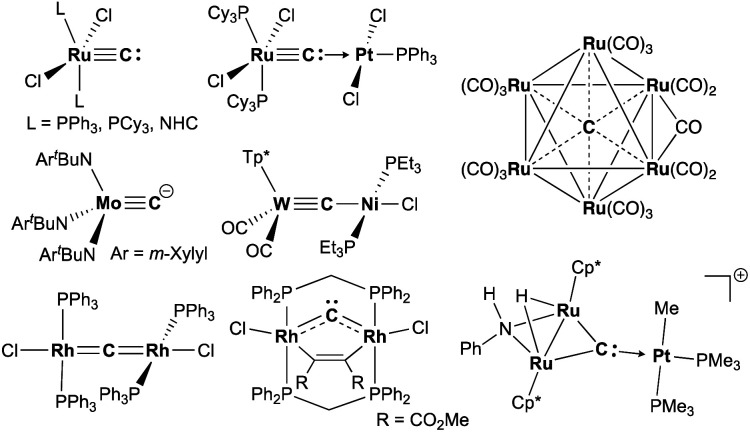
Examples of metal carbido complexes: neutral terminal carbide, anionic terminal carbide, dative bonding μ-carbide, metallocarbyne, dimetallacumulene, dimetallocarbene, trinuclear μ_3_-carbide, and interstitial carbide.

Carbide is a special ligand in organometallic,^1–3^ materials,^4–6^ and bioinorganic chemistry,^7–9^*i.e.* the core structure in nitrogenases.^7^ Despite numerous studies on stable interstitial carbido clusters^2,3,10–13^ and carbido materials^4–6^ as well as gas-phase generation of metal carbides,^14–16^ examples of more exposed and reactive molecular metal carbides, such as mononuclear, dinuclear, or trinuclear complexes, are still rare.^1^ As shown in Fig. 1, several types of molecular metal carbido species have been reported. Terminally bound carbide species can be categorized into two types based on their electronic structures. The neutral carbido complexes [(L)_2_Cl_2_M≡C:] (M = Ru,^17–19^ Os;^20^ L = PPh_3_, PCy_3_, NHC), originally synthesized by Heppert in 2002,^17^ can form dative bonding Lewis pairs with various transition metals.^21–25^ The anionic terminal carbide [(Ar^*t*^BuN)_3_Mo≡C]^−^ (Ar = *m*-Xylyl) has been prepared by Cummins and co-workers *via* deprotonation of a terminal methylidyne complex.^26^ Akin to the neutral terminal carbides,^17–21^ the anionic carbides also bind various metals to form dinuclear structures, but those dinuclear carbido complexes have a metallocarbyne character.^27–31^ In addition to those two types of dinuclear carbido complex, dimetallacumulene structures of dinuclear μ-carbides have been reported generally with late-transition metals.^32–38^ Recently, Hill and co-workers have reported a bent dimetallacumulene, which has a rather dimetallocarbene character, called dirhoda-heterocyclic carbene.^39^ The dimetallocarbene species^39,40^ can bind various Lewis acids to form trinuclear μ_3_-carbides, which have been investigated by Takemoto and Matsuzaka.^41,42^

Carbido complexes have been generally prepared by deprotonation of methylidynes,^26,40,42^ metallation of halocarbynes,^28,43^ or multiple C–X bond cleavages of various carbide sources, such as CO,^10,44–46^ CI_4_,^32^ CHCl_3_,^35^ and CS_2_.^36,37^ Although functionalization of lithiocarbynes [(OC)_2_(Tp*)M≡CLi(thf)_*n*_] (M = Mo, W; Tp* = tris(3,5-dimethyl-1-pyrazolyl)borate), prepared by lithiation of bromocarbynes [(OC)_2_(Tp*)M≡CBr],^43^ has been widely explored by Hill and co-workers,^43,47–50^ reactivity studies on carbide transfer of carbido ligands as a C1 source have not been developed. Herein, we report reduction of CBr_4_ with CrBr_2_ and structural characterization of a chromium carbide as well as a reactivity study for carbide transfer.

## Results and discussion

### Halocarbyne and carbide transfer to aldehydes by the CX_4_–CrX_2_ reagent (X = Cl, Br)

**Scheme 1:**
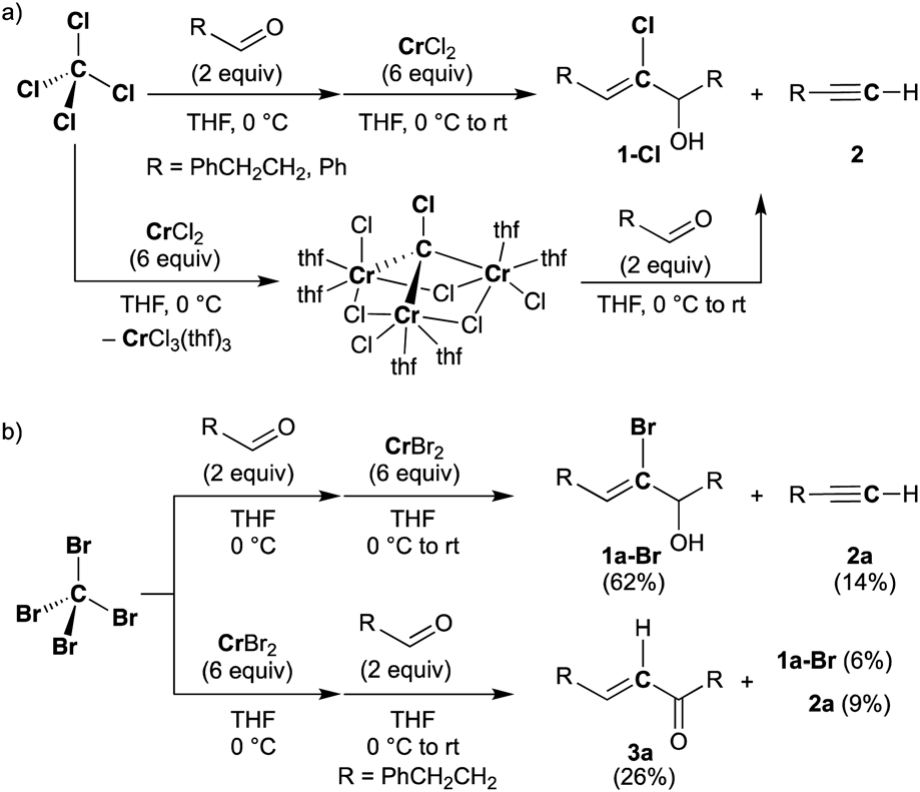
Reactions of aldehydes with the CX_4_–CrX_2_ reagent ((a) X = Cl, (b) X = Br).

Falck and Mioskowski have reported a chlorocarbyne transfer reaction to aldehydes by treatment with a mixture of CCl_4_ and CrCl_2_ in a 1 : 6 ratio.^51^ Recently, we have achieved characterization of a trinuclear chromium chlorocarbyne complex, [CrCl(thf)_2_]_3_(μ_3_-CCl)(μ-Cl)_3_, obtained from the CCl_4_–CrCl_2_ reagent,^52^ and the chlorocarbyne complex indeed underwent chlorocarbyne transfer to aldehydes to afford chloroallylic alcohols **1-Cl** (Scheme 1a). In addition to formation of **1-Cl**, Corey–Fuchs-type homologation products **2**,^53^ terminal alkynes, were also formed *via* Cl-abstraction as a formal carbide transfer from the chlorocarbyne complex. A bromocarbyne transfer reaction has also been reported for the bromide analogues CBr_4_ and CrBr_2_,^51^ which was prepared *in situ* by reduction of CrBr_3_ with LiAlH_4_. We have revisited the classical protocol of preparation of CrBr_2_ by treatment of chromium(0) with hydrobromic acid.^54^ Although the Cr^2+^ ion is still fairly reducing (Cr^3+^/Cr^2+^: −0.424 V *vs.* SHE), a blue solid of the chromium(ii) bromide hydrate [CrBr_2_(H_2_O)_6_] was readily precipitated out from an aqueous mixture of chromium(0) powder and hydrobromic acid at 0 °C.^55^ Having a pure solid of anhydrous CrBr_2_ in hand, we demonstrated the bromocarbyne transfer to aldehydes by use of the isolated CrBr_2_. To our surprise, in addition to formation of the bromoallylic alcohol **1a-Br** and terminal alkyne **2a**, an α,β-unsaturated ketone **3a** was also formed (Scheme 1b). Interestingly, pre-mixing CBr_4_ and CrBr_2_ in THF prior to treatment with aldehydes resulted in the formation of the α,β-unsaturated ketone **3a** as a major product. Unfortunately, formation of unidentifiable products by further reactions of **3a** with THF^56,57^ promoted by some low-valent chromium species lowered the yields of **3a**. Combinations of different halogens CCl_4_–CrBr_2_ and CBr_4_–CrCl_2_ were also attempted, but a mixture of all four products **1a-Cl**, **1a-Br**, **2a**, and **3a** was formed in both cases due to halogen-scrambling.

### Reduction of CBr_4_ with CrBr_2_

**Scheme 2:**
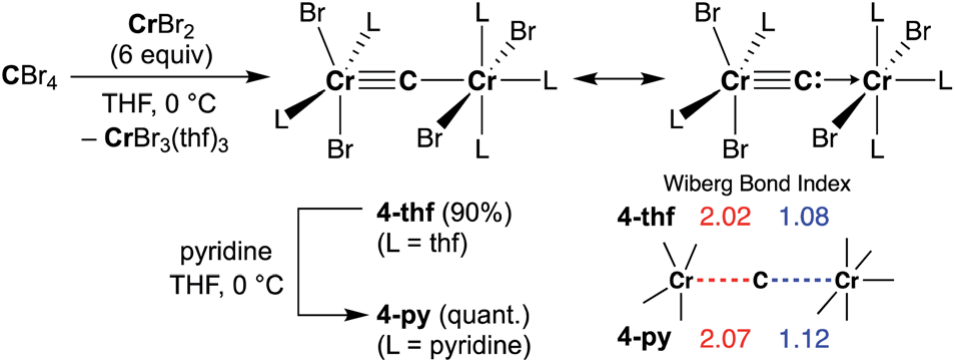
Synthesis of **4-thf** by reduction of CBr_4_ with CrBr_2_ and ligand-exchange with pyridine to form **4-py**.

**Fig. 2:**
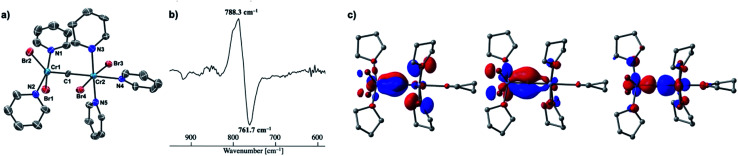
(a) Solid-state structure of **4-py** with thermal ellipsoids at the 50% probability level. Hydrogen atoms and solvated THF molecules have been omitted for clarity; (b) difference IR spectrum of **4-thf** and **4-13C** (**4-thf**: positive, **4-13C**: negative); (c) molecular orbitals (isovalue = 0.05) of **4-thf** representing Cr–C bonding interactions (left and middle: π-interactions, right: σ-interaction).

Given the idea of formation of the α,β-unsaturated ketone **3a**, we inquired if the more reactive tetrahalomethane CBr_4_ could be further reduced by chromium(ii) to form a chromium carbide species rather than a bromocarbyne species. Akin to the reduction of CCl_4_ with CrCl_2_ recently reported by our group,^52^ treatment of CBr_4_ with 6 equivalents of CrBr_2_ in THF at 0 °C produced [CrBr_3_(thf)_3_]^58^ as a brown solid along with a green supernatant. After removal of [CrBr_3_(thf)_3_], a green solid was obtained from the green supernatant and identified as paramagnetic **4-thf** in 90% yield (Scheme 2). The green product **4-thf** is stable in the solid state at room temperature, but slightly unstable in solution even at low temperature (−78 °C) to form [CrBr_3_(thf)_3_] and some unidentified chromium species. An XRD study of **4-thf** revealed a dinuclear structure, namely [CrBr_2_(thf)_2_)][CrBr_2_(thf)_3_](μ-C), where a carbido ligand is bridging between 5- and 6-coordinate chromium centers (Fig. S35†). Upon crystallization of **4-thf** to obtain a better crystal for the XRD study, complex **4-thf** gradually decomposed in solution to form a precipitate of [CrBr_3_(thf)_3_]. The conclusive structural characterization was performed with a pyridine adduct, **4-py**, which was obtained quantitatively by addition of pyridine to **4-thf** in THF. The molecular structure of **4-py** (Fig. 2a) still maintains a dinuclear chromium moiety bridged by a carbido ligand in a linear fashion (Cr1–C1–Cr2 = 174.5(6)°). The 5-coordinate chromium center is best described as distorted square pyramidal (*τ*_5_ = 0.29)^59^ with the carbido ligand on the apical position along with a short Cr1–C1 bond (1.634(10) Å), while the other chromium center represents a distorted octahedral geometry with a long Cr2–C1 bond (2.035(10) Å). Akin to the reported mononuclear neutral carbides and dinuclear dative bonding carbides, two X-type ligands (halides) and two L-type ligands (THF, pyridine, PR_3_ or NHC) are transoid to each other on the basal positions in the 5-coordinate environment, but the *τ*_5_ values of **4-thf** (*τ*_5_ = 0.41) and **4-py** (*τ*_5_ = 0.29) are larger than those of the mononuclear neutral carbides (*τ*_5_ = 0.07–0.16)^17–20^ and dinuclear dative bonding carbides (*τ*_5_ = 0.01–0.23)^21–25^ of ruthenium and osmium. Given the *S* = 2 nature of **4-thf** in THF by Evans' method (*μ*_eff_ = 4.84 *μ*_B_)^60^ as well as in the solid state (*μ*_eff_ = 5.03),^55^ the Wiberg bond indices of Cr1–C1 and Cr2–C1 bonds were calculated as 2.02 and 1.08, respectively, for **4-thf** and 2.07 and 1.12 for **4-py**. Bonding analyses of the neutral terminal carbides [(Me_3_P)_2_Cl_2_M≡C:] (M = Fe, Ru) have been previously reported by Krapp, Pandey, and Frenking.^61^ Although the Wiberg bond indices were *ca.* 2 for the M–C bond in the neutral terminal carbides, an M≡C triple bond character has been shown with two π- and one σ-type bonding orbitals. Molecular orbitals of **4-thf** as well as **4-py** also depicted two π-bonding interactions of the carbido ligand more delocalized around the 5-coordinate chromium center and a three-center two-electron [Cr–C–Cr] σ-interaction (Fig. 2c). Therefore, the canonical structure of the carbido complex **4** could be better described as a dative bonding μ-carbide^21–25^ than the metallocarbyne character.^27–31^

**Scheme 3:**
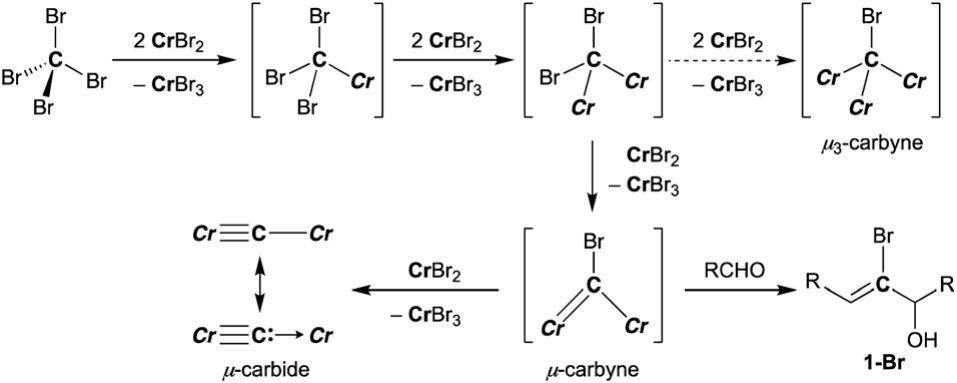
Reduction of C–Br bonds with CrBr_2_ and formation of **4-thf** (*Cr* = CrBr_2_(thf)_*n*_).

A plausible pathway of formation of the carbido complex **4-thf** from CBr_4_ and CrBr_2_ is shown in Scheme 3. Akin to other Cr–C bond formations by reduction of haloalkanes with chromium(ii) halides,^62–66^ single-electron reductions of C–Br bonds and subsequent radical coupling should proceed to form Cr–C bonds in the CBr_4_–CrBr_2_ system. As shown in Schemes 1b and 3, the transient bromocarbyne species could be trapped by aldehydes to afford the bromoallylic alcohols **1-Br**.^51^ However, as far as we have attempted to isolate the bromocarbyne intermediate, no chromium species other than the carbido complex **4-thf** and [CrBr_3_(thf)_3_] could be obtained by limiting the stoichiometry of CrBr_2_ to CBr_4_ or controlling the reaction temperature. Although the trinuclear μ_3_-chlorocarbyne complex [CrCl(thf)_2_]_3_(μ_3_-CCl) (μ-Cl)_3_ was obtained from the CCl_4_–CrCl_2_ system,^52^ a similar trinuclear framework bridged by bromides may be difficult for the bromocarbyne ligand to bridge in a μ_3_-fashion due to a larger ionic radius of bromine. Therefore, the transient dinuclear bromocarbyne could be further reduced by CrBr_2_ to cleave the last C–Br bond rather than forming a μ_3_-carbyne scaffold bridged by bromides, resulting in the formation of the dinuclear carbido complex **4-thf**.

### Isotope labeling studies

**Scheme 4:**
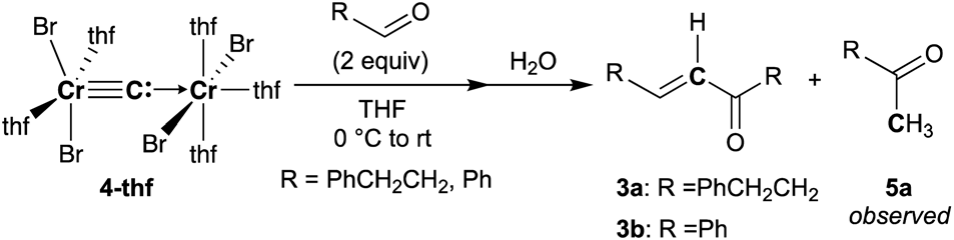
Reactions of **4-thf** with aldehydes.

**Scheme 5:**
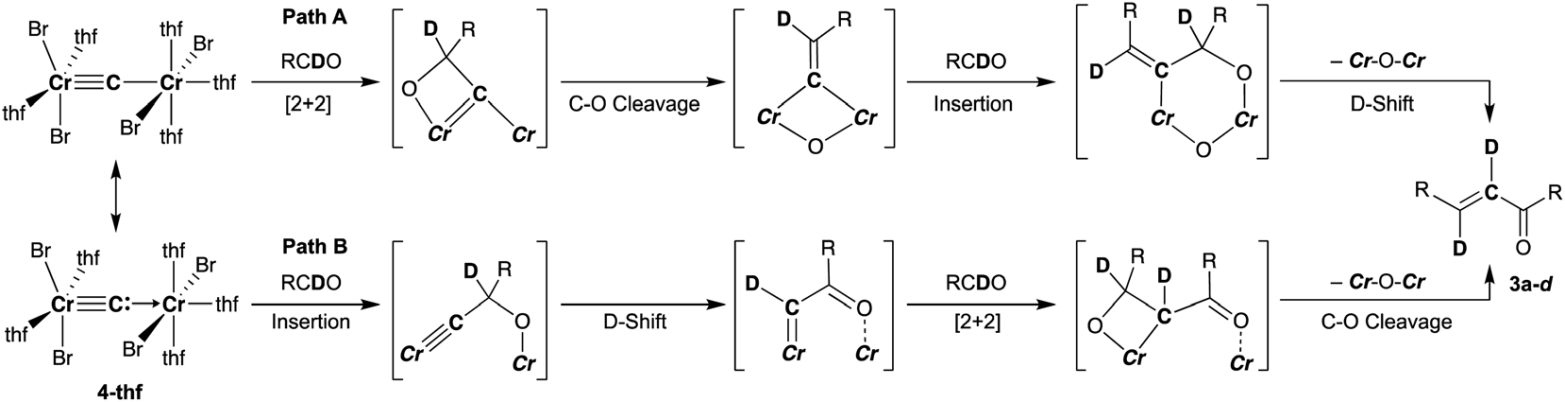
Two plausible pathways to afford the α,β-unsaturated ketone **3** by reaction of **4-thf** with aldehyde based on the isotope labeling studies.

To spectroscopically confirm the carbido ligand delivered from CBr_4_, the isotopologue [CrBr_2_(thf)_2_)][CrBr_2_(thf)_3_](μ-^13^C) (**4-13C**) was prepared from ^13^CBr_4_. An IR spectrum of **4-13C** revealed an absorption of the [Cr–C–Cr] 3-centered vibration at 762 cm^−1^, which was red-shifted from 788 cm^−1^ observed in the unlabeled carbide **4-thf** (Fig. 2b). Measurement of a ^13^C NMR spectrum for **4-13C** was also attempted, but ^13^C NMR signals other than solvents could not be located probably due to the paramagnetic feature of the chromium carbide. Akin to the *in situ* preparation of the CBr_4_–CrBr_2_ reagent, the isolated carbido complex **4-thf** readily reacted with 2 equivalents of aldehydes (Scheme 4) to form the α,β-unsaturated ketones **3a** (22%) and **3b** (20%).^55^ Accordingly, a ^13^C-labeled α,β-unsaturated ketone **3a-13C** was also prepared by use of **4-13C**. The NMR spectrum of **3a-13C** displayed an enriched ^13^C NMR signal selectively on the α-position at 130.85 ppm, which was coupled with α-H at 6.10 ppm (^1^*J*_CH_ = 157 Hz). The deuterium labeling study was also demonstrated using a deuterated aldehyde, 3-phenylpropanal-*d*. The ^2^H NMR spectrum of **3a–d** displayed deuterium signals on both α- and β-positions at 6.15 ppm and 6.88 ppm, respectively, implying that some H-shift event took place from the aldehyde to the α-carbon of the α,β-unsaturated ketone, which was delivered from the carbido ligand. As illustrated in Scheme 5, two plausible pathways to give the α,β-unsaturated ketones **3** could be considered from two canonical structures of **4-thf**. Path A shows [2 + 2]-cycloaddition of the first aldehyde to the Cr≡C bond, while Path B represents insertion of the aldehyde into the Cr–C or dative bond. Analyses of the quenched reaction mixture of aldehydes with **4-thf** as well as the pre-mixed CBr_4_–CrBr_2_ revealed the formation of ketone **5a**. The ketone **5a** could be formed by hydrolysis of one of the intermediates represented in Path B, implying that the insertion pathway B is the more likely pathway.^67^

### Double-cyclopropanation by complex **4-thf** to alkenes

**Scheme 6:**
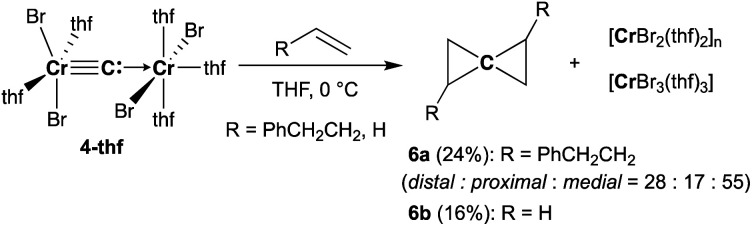
Double-cyclopropanation by complex **4-thf** to alkenes.

**Fig. 3:**
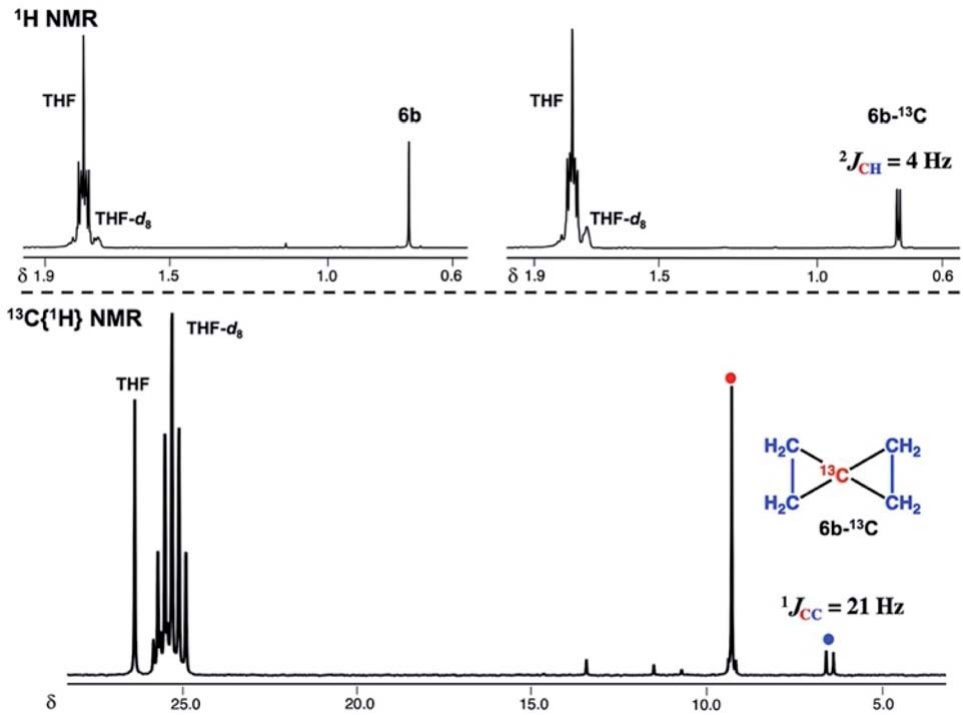
Top: ^1^H NMR spectra of **6b** (left) and **6b-13C** (right). Bottom: ^13^C{^1^H} NMR spectrum of **6b-13C**.

To gain further insight into the electronic structure of the dinuclear carbide **4**, we hypothesized that a reactivity study of **4-thf** with unsaturated substrates, such as alkenes or alkynes, would give some idea about which canonical structure of **4-thf** is more dominant. A terminal alkene, 4-phenyl-1-butene, gradually reacted with **4-thf**, resulting in precipitation of a mixture of [CrBr_2_(thf)_2_]_*n*_ ^68^ and [CrBr_3_(thf)_3_] (Scheme 6). After work-up, analyses by NMR and GC-MS spectroscopy revealed formation of spiropentane **6a** (24% isolated yield) as a mixture of diastereomers^69^ (distal : proximal : medial = 28 : 17 : 55),^55^ and the spiropentane **6a** was also prepared in 22% yield (distal : proximal : medial = 32 : 20 : 48) by the CBr_4_–CrBr_2_ reagent prepared *in situ* (Fig S15†). Addition of ethylene (1 atm) to a THF solution of **4-thf** resulted in the formation of the parent spiropentane (**6b**). To conclusively confirm the formation of spiropentanes **6a** and **6b** by carbide transfer from **4-thf**, the ^13^C-enriched carbide **4-13C** was treated with 4-phenyl-1-butene and ethylene, respectively. As a result, the quaternary carbons in the spiropentane skeleton were indeed ^13^C-enriched in the ^13^C NMR spectra (Fig. 3) of **6a-13C** (19.51 ppm, 21.19 ppm, and 21.22 ppm) and **6b-13C** (9.30 ppm).

Spiropentane^70,71^ is still a challenging framework to assemble by organic synthetic methods such as reduction of tetrakis(1-haloalkyl)methanes^72,73^ due to multiple side-reactions and isomerization. Formal “double-cyclopropanation” by a carbido moiety to alkenes has been reported in the gas phase by use of carbon vapor,^74,75^ which was generated by arc discharge, but isomerization of the resulting spiropentanes also proceeded under such harsh conditions. Although the yields of the obtained spiropentanes **6a** and **6b** are still low, the carbido complex **4-thf** underwent “double-cyclopropanation” to alkenes similar to the carbon vapor but without isomerization.

### Synthesis of cyclopropenylidene complexes

**Scheme 7:**
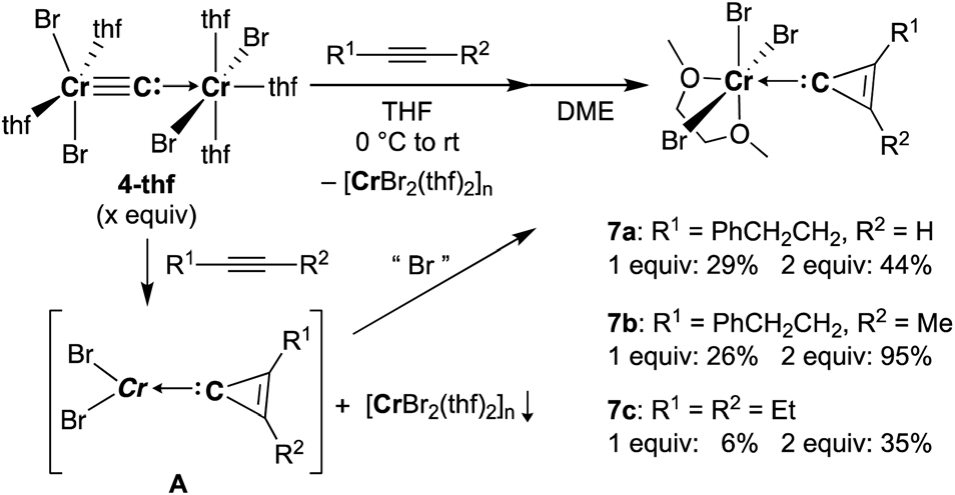
Synthesis of cyclopropenylidene complexes.

**Fig. 4:**
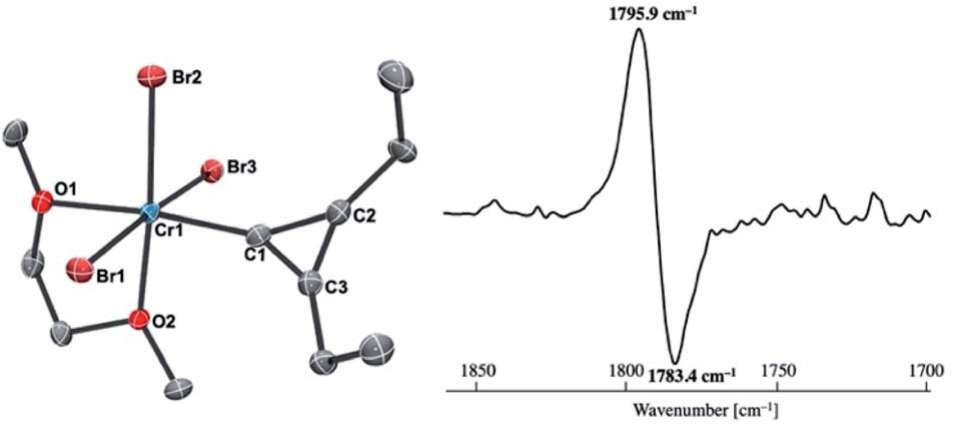
(left) Solid-state structure of complex **7c** with thermal ellipsoids at the 50% probability level. Hydrogen atoms have been omitted for clarity. (right) Difference IR spectrum (**7c**: positive, **7c-13C**: negative).

Reaction of **4-thf** with alkynes smoothly proceeded to form a precipitate of [CrBr_2_(thf)_2_]_*n*_. After removal of [CrBr_2_(thf)_2_]_*n*_, paramagnetic green products **7a** and **7b** were obtained from a DME solution (Scheme 7). A single crystal suitable for an XRD study was obtained by crystallization of the 3-hexyne analogue **7c** (*μ*_eff_ = 3.69 *μ*_B_) from a DME solution layered with hexane. As shown in Fig. 4, a solid-state structure of **7c** displayed a cyclopropenylidene ligand on a chromium(iii) bromide [*mer*-CrBr_3_(dme)] along with a typical Cr(iii)–carbene dative bond (2.039(3) Å).^76–78^ Akin to the reported chromium cyclopropenylidene complexes^79–81^ as well as the free cyclopropenylidenes,^82,83^ the cyclopropenylidene complex **7c** also has a slightly shorter C–C bond (C2–C3: 1.351(4) Å) on the backbone than the other C–C bonds (C1–C2: 1.383(4) Å, C1–C3: 1.395(4) Å) in the cyclopropenylidene unit, implying delocalization of p-electrons over the 3-membered ring. IR spectra revealed a characteristic absorption of the cyclopropenylidene unit at 1796 cm^−1^, which was red-shifted to 1783 cm^−1^ by the ^13^C-enriched cyclopropenylidene **7c-13C**. In contrast to the similar IR absorption observed in **7b**/**7b-13C** at 1809/1796 cm^−1^, the IR spectra of the mono-substituted cyclopropenylidene complexes **7a**/**7a-13C** delivered from the terminal alkyne showed two absorptions of the cyclopropenylidene ligand at 1351/1325 cm^−1^ and 1740/1720 cm^−1^ (Fig. S27†).

Despite the tetrabromide complex **4-thf** reacting with alkynes, a mixture of tribromide **7** and dibromide [CrBr_2_(thf)_2_]_*n*_ was obtained. Thus, the third bromide in **7** could be provided by disproportionation of the resulting chromium(ii) cyclopropenylidene **A** or another equivalent of carbide **4**. Although the source of bromide in the formation of **7** is still unclear, use of two equivalents of carbide **4** to alkynes dramatically improved the yields of cyclopropenylidene complexes **7a** (1 equiv.: 29% yield; 2 equiv.: 44% yield), **7b** (1 equiv.: 26% yield; 2 equiv.: 95% yield), and **7c** (1 equiv.: 6% yield; 2 equiv.: 35% yield). Note that addition of [CrBr_3_(thf)_3_] into the reaction was also attempted, but the yields of the cyclopropenylidene complexes were not improved.

Formation of a cyclopropenylidene unit by reaction of the ruthenium carbido complex [(Ph_3_P)_2_Cl_2_Ru≡C:] with alkyne has been reported by Johnson and co-workers.^84^ The ruthenium carbide reacted only with an electron-deficient alkyne, dimethylacetylene dicarboxylate (DMAD), to form a cyclopropenylidene complex. In contrast to the ruthenium carbide [(Ph_3_P)_2_Cl_2_Ru≡C:], our chromium carbide **4-thf** reacted with electron-rich alkynes to form cyclopropenylidene complexes **7a**, **7b**, and **7c**, while no reaction of **4-thf** with DMAD in THF was observed along with a gradual decomposition of unreacted **4-thf** in solution. Addition of alkenes as well as DMAD to cyclopropenylidene complexes **7a**, **7b**, and **7c** has also been attempted, but identifiable products could not be obtained.

## Conclusions

This work has shown that the CBr_4_–CrBr_2_ reagent possesses bromocarbyne and carbide transfer abilities to aldehydes. In the CBr_4_–CrBr_2_ reagent, the first example of a chromium carbido complex was isolated and structurally characterized. DFT calculations and NBO analyses of the carbido complex proposed a dative bonding μ-carbide character. Reactivity studies on carbide transfer of the carbido ligand with aldehydes and alkenes as well as isotope labeling studies have been demonstrated. It is noteworthy that a formal double-cyclopropanation of the carbido complex to alkenes might support the dative bond character of the carbido moiety. In addition, rare examples of cyclopropenylidene complexes have been prepared by treatment of the carbido complex with terminal and internal alkynes. Further investigation of carbide transfer is in progress.

## Data availability

All experimental data, NMR spectra, FT-IR spectra, UV-Vis spectra, GC-MS data, crystallographic data, and computational analyses are provided in the ESI.†

## Author contributions

K. Irifune prepared compounds and carried out reactions. T. Kurogi performed spectroscopy, crystallography, and computational studies and analyzed the data. T. Kurogi and K. Takai supervised this study and wrote the manuscript. All authors discussed the results and contributed to the peparation of the final manuscript.

## Conflicts of interest

There are no conflicts to declare.

## Supplementary Material

SC-012-D1SC04910K-s001

SC-012-D1SC04910K-s002

## References

[cit1] Takemoto S., Matsuzaka H. (2012). Coord. Chem. Rev..

[cit2] Johnson B. F. G., Lewis J., Nelson W. J. H., Nicholls J. N., Vargas M. D. (1983). J. Organomet. Chem..

[cit3] Tachikawa M., Muetterties E. L. (1981). Prog. Inorg. Chem..

[cit4] Xiao Y., Hwang J.-Y., Sun Y.-K. (2016). J. Mater. Chem. A.

[cit5] Anasori B., Lukatskaya M. R., Gogotsi Y. (2017). Nat. Rev. Mater..

[cit6] Sun J., Zhao J., Huang Z., Yan K., Shen X., Xing J., Gao Y., Jian Y., Yang H., Li B. (2020). Nano-Micro Lett..

[cit7] Lancaster K. M., Roemelt M., Ettenhuber P., Hu Y., Ribbe M. W., Neese F., Bergmann U., DeBeer S. (2011). Science.

[cit8] Wiig J. A., Lee C. C., Hu Y., Ribbe M. W. (2013). J. Am. Chem. Soc..

[cit9] Grunenberg J. (2017). Angew. Chem., Int. Ed..

[cit10] Johnson B. F. G., Johnson R. D., Lewis J. (1968). J. Chem. Soc. A.

[cit11] Scherbaum F., Grohmann A., Huber B., Krüger C., Schmidbaur H. (1988). Angew. Chem., Int. Ed. Engl..

[cit12] Scherbaum F., Grohmann A., Müller G., Schmidbaur H. (1989). Angew. Chem., Int. Ed. Engl..

[cit13] Daugherty N. T., Robilotto T. J., Bacsa J., Gray T. G., Sadighi J. P. (2020). Polyhedron.

[cit14] Cassady C. J., McElvany S. W. (1990). J. Am. Chem. Soc..

[cit15] Zhang R., Dinca A., Fisher K. J., Smith D. R., Willett G. D. (2005). J. Phys. Chem. A.

[cit16] Sabor S., Benjelloun A. T., Mogren M. M. A., Mochlaf M. (2014). Phys. Chem. Chem. Phys..

[cit17] Carlson R. G., Gile M. A., Heppert J. A., Mason M. H., Powell D. R., Velde D. V., Vilain J. M. (2002). J. Am. Chem. Soc..

[cit18] Hejl A., Trnka T. M., Day M. W., Grubbs R. H. (2002). Chem. Commun..

[cit19] Morsing T. J., Reinholdt A., Sauer S. P. A., Bendix J. (2016). Organometallics.

[cit20] Stewart M. H., Johnson M. J. A., Kampf J. W. (2007). Organometallics.

[cit21] Hong S. H., Day M. W., Grubbs R. H. (2004). J. Am. Chem. Soc..

[cit22] Reinholdt A., Vibenholt J. E., Morsing T. J., Schau-Magnussen M., Reeler N. E. A., Bendix J. (2015). Chem. Sci..

[cit23] Reinholdt A., Herbst K., Bendix J. (2016). Chem. Commun..

[cit24] Reinholdt A., Bendix J. (2017). Inorg. Chem..

[cit25] Reinholdt A., Hill A. F., Bendix J. (2018). Chem. Commun..

[cit26] Peters J. C., Odom A. L., Cummins C. C. (1997). Chem. Commun..

[cit27] Latesky S. L., Selegue J. P. (1987). J. Am. Chem. Soc..

[cit28] Etienne M., White P. S., Templeton J. L. (1991). J. Am. Chem. Soc..

[cit29] Cade I. A., Hill A. F., McQueen C. M. A. (2009). Organometallics.

[cit30] Frogley B. J., Hill A. F. (2019). Chem. Commun..

[cit31] Burt L. K., Hill A. F. (2020). Dalton Trans..

[cit32] Mansuy D., Lecomte J. P., Chottard J. C., Bartoli J. F. (1981). Inorg. Chem..

[cit33] Kienast A., Bruhn C., Homborg H. (1997). Z. Anorg. Allg. Chem..

[cit34] Galich L., Kienast A., Hüchstädt H., Homborg H. (1998). Z. Anorg. Allg. Chem..

[cit35] Colomban C., Kudrik E. V., Tyurin D. V., Albrieux F., Nefedov S. E., Afanasiev P., Sorokin A. B. (2015). Dalton Trans..

[cit36] Young R. D., Hill A. F., Cavigliasso G. E., Stranger R. (2013). Angew. Chem., Int. Ed..

[cit37] Kalläne S. I., Braun T., Teltewskoi M., Braun N., Herrmann R., Laubenstein R. (2015). Chem. Commun..

[cit38] Barnett H. J., Hill A. F. (2020). Chem. Commun..

[cit39] Barnett H. J., Hill A. F. (2020). Angew. Chem., Int. Ed..

[cit40] Takemoto S., Ohata J., Umetani K., Yamaguchi M., Matsuzaka H. (2014). J. Am. Chem. Soc..

[cit41] Takemoto S., Morita H., Karitani K., Fujiwara H., Matsuzaka H. (2009). J. Am. Chem. Soc..

[cit42] Takemoto S., Tsujita M., Matsuzaka H. (2017). Organometallics.

[cit43] Cordiner R. L., Hill A. F., Wagler J. (2008). Organometallics.

[cit44] Caselli A., Solari E., Scopelliti R., Floriani C. (2000). J. Am. Chem. Soc..

[cit45] Miller R. L., Wolczanski P. T. (1993). J. Am. Chem. Soc..

[cit46] Buss J. A., Agapie T. (2016). Nature.

[cit47] Colebatch A. L., Hill A. F. (2016). Organometallics.

[cit48] Hill A. F., Shang R. (2012). Organometallics.

[cit49] Colebatch A. L., Hill A. F., Shang R., Wills A. C. (2010). Organometallics.

[cit50] Colebatch A. L., Frogley B. J., Hill A. F., Onn C. S. (2021). Chem.–Eur. J..

[cit51] Baati R., Barma D. K., Falck J. R., Mioskowski C. (2002). Tetrahedron Lett..

[cit52] Kurogi T., Irifune K., Enoki T., Takai K. (2021). Chem. Commun..

[cit53] Corey E. J., Fuchs P. L. A. (1972). Tetrahedron Lett..

[cit54] Holah D. G., Fackler, Jr J. P. (1967). Inorg. Synth..

[cit55] See the ESI†

[cit56] Lan Y., Fan P., Liu X.-W., Meng F.-F., Ahmad T., Xu Y.-H., Loh T.-P. (2017). Chem. Commun..

[cit57] Lee G. S., Hong S. H. (2018). Chem. Sci..

[cit58] Jones P. J., Hale A. L., Levason W., McCullough, Jr F. P. (1983). Inorg. Chem..

[cit59] Addison A. W., Rao T. N., Reedijk J., van Rijn J., Verschoor G. C. (1984). J. Chem. Soc., Dalton Trans..

[cit60] Evans D. F. (1959). J. Chem. Soc..

[cit61] Krapp A., Pandey K. K., Frenking G. (2007). J. Am. Chem. Soc..

[cit62] Anet F. A. L., Leblanc E. (1957). J. Am. Chem. Soc..

[cit63] Anet F. A. L. (1959). Can. J. Chem..

[cit64] Castro C. E., Kray, Jr W. C. (1963). J. Am. Chem. Soc..

[cit65] Kochi J. K., Davis D. D. (1964). J. Am. Chem. Soc..

[cit66] Castro C. E., Kray, Jr W. C. (1966). J. Am. Chem. Soc..

[cit67] Preliminary studies of the first steps of Paths A and B, [2 + 2]-cycloaddition of C=O to Cr≡C and insertion of aldehyde into Cr–C, respectively, with the help of DFT calculations showed that the intermediate formed by the first insertion reaction in Path B (+12.2 kcal mol^−1^) was much more stable than the [2 + 2]-cycloaddition product in Path A (+33.6 kcal mol^−1^) compared with Δ*G*(sol) values from **4-thf** and PhCHO of 0 kcal mol^−1^. However, disproportionation of chromium species can also be considered during the reaction with aldehydes as observed in the reaction of **4-thf** with alkynes, and it hampered mechanistic studies of these two reaction pathways

[cit68] Twamley B., Zehnder R., Shapiro P. J. (2001). Acta Crystallogr., Sect. E: Struct. Rep. Online.

[cit69] Guan H.-P., Ksebati M. B., Cheng Y.-C., Drach J. C., Kern E. R., Zemlicka J. (2000). J. Org. Chem..

[cit70] Donahue J., Humphrey G. L., Schomaker V. (1945). J. Am. Chem. Soc..

[cit71] Murray M. J., Stevenson E. H. (1944). J. Am. Chem. Soc..

[cit72] Murray M. J., Stevenson E. H. (1944). J. Am. Chem. Soc..

[cit73] Applequist D. E., Fanta G. F., Henrikson B. W. (1958). J. Org. Chem..

[cit74] Skell P. S., Engel R. R. (1965). J. Am. Chem. Soc..

[cit75] Skell P. S., Enge R. R. (1966). J. Am. Chem. Soc..

[cit76] Malan F. P., Singleton E., van Rooyen P. H., Conradie J., Landman M. (2018). New J. Chem..

[cit77] Kreisel K. A., Yap G. P. A., Theopold K. H. (2006). Organometallics.

[cit78] McGuinness D. S., Gibson V. C., Wass D. F., Steed J. W. (2003). J. Am. Chem. Soc..

[cit79] Öfele K. (1968). Angew. Chem., Int. Ed..

[cit80] Huttner G., Schelle S., Mills O. S. (1969). Angew. Chem., Int. Ed..

[cit81] Juneau K. N., Hegedus L. S., Roepke F. W. (1989). J. Am. Chem. Soc..

[cit82] Lavallo V., Canac Y., Donnadieu B., Schoeller W. W., Bertrand G. (2006). Science.

[cit83] Holschumacher D., Hrib C. G., Jones P. G., Tamm M. (2007). Chem. Commun..

[cit84] Caskey S. R., Stewart M. H., Johnson M. J. A., Kampf J. W. (2006). Angew. Chem., Int. Ed..

