# Supplementary material for: Chromium carbides and cyclopropenylidenes
Source: Chem Sci. 2021 Oct 8;12(42):14281–7. doi: 10.1039/d1sc04910k (PMC8565369; doi:10.1039/d1sc04910k)
Supplement: SC-012-D1SC04910K-s001 [file SC-012-D1SC04910K-s001.pdf]

Electronic Supplementary Material (ESI) for Chemical Science.
This journal is © The Royal Society of Chemistry 2021

Chromium carbides and cyclopropenylidenes

Takashi Kurogi,[†, ‡,*] Keiichi Irifune,[†] and Kazuhiko Takai[†,*]

[†]*Division of Applied Chemistry, Graduate School of Natural Science and Technology, Okayama University, 3-1-1 Tsushimanaka, Kita-ku, Okayama 700-8530, Japan.*
[‡]Present address: *Department of Chemistry, Graduate School of Science, Kyoto University, Kitashirakawaoiwake-cho, Sakyo-ku, Kyoto 606-8502, Japan.*

Table of Contents

**General Procedure**	S3
**Synthesis of $CrBr_2$**	S4-S5
**Synthesis of $[CrBr_2(thf)_2)][CrBr_2(thf)_3)](\mu\text{-}C)$ (4-thf)**	S6-S8
**Synthesis of $[CrBr_2(thf)_2)][CrBr_2(thf)_3)](\mu\text{-}^{13}C)$ (4-^{13}C)**	S8-S9
**Reaction of 3-Phenylpropanal with $CrBr_2$ and CBr_4**	S10-S13
**Reaction of 3-Phenylpropanal with 4-thf**	S13
**Reaction of Benzaldehyde with 4-thf**	S13-S14
**Reaction of 3-Phenylpropanal with 4-^{13}C**	S14-S16
**Reaction of 3-Phenylpropanal-*d* with 4-thf**	S16-S17
**Reaction of 4-Phenyl-1-butene with 4-thf**	S18-S20
**Reaction of 4-Phenyl-1-butene with 4-^{13}C**	S21-S22
**Reaction of Ethylene with 4-thf**	S22-S23
**Reaction of Ethylene with 4-^{13}C**	S24-S25
**Synthesis of 7a**	S25-S26
**Synthesis of 7b**	S27-S28
**Synthesis of 7c**	S28-S29
**Synthesis of 7a-^{13}C**	S30-S31
**Synthesis of 7b-^{13}C**	S31-S32
**Synthesis of 7c-^{13}C**	S33-S34
**FID-GC and GC-MS Analyses**	S35-S38
**X-ray Crystallography**	S39-S40

**Crystallographic data of 4-py**	S41
**Crystallographic data of 7c**	S42
**Computational Details**	S43-S44
**Cartesian Coordinates of the Optimized Geometries**	S45-S48
**References**	S49

General Procedure

All operations were performed in a Vac glove box under a dinitrogen atmosphere or using standard Schlenk techniques under an argon atmosphere unless otherwise stated. Anhydrous hydrocarbon solvents (pentane, hexane), stabilizer-free THF, and pyridine were purchased from FUJIFILM Wako Pure Chemical Corporation. Anhydrous DME was purchased from Kanto Chemical Corporation. All anhydrous solvents were stored over activated 4 Å molecular sieves and degassed by bubbling argon. Molecular sieves were activated under vacuum at 200 °C. Chromium(0) powder (99.9%) was purchased from Kojundo Chemical Laboratory Corporation. Carbon tetrabromide (99%) was purchased from TCI and purified by sublimation with Kugelrohr. Benzaldehyde and 3-phenylpropanal were purified by distillation prior to use. Ethylene gas (99.9%) was purchased from Nippon Sanso Holding Corporation. Chloroform-*d* (99.8 atom% D) and THF-d_8 (99.5 atom% D) were purchased from Cambridge Isotope Laboratories. ^{13}C-Enriched carbon tetrabromide (99.0 atom% ^{13}C) was purchased from Santa Cruz Bio Tech. 5-Phenyl-2-pentyne was prepared according to the reported procedure.[1] All other chemicals were purchased from commercial sources. ^{1}H, ^{13}C and HMQC NMR spectra were recorded on a JEOL ECS 400 MHz spectrometer. ^{1}H and ^{13}C NMR chemical shifts are reported referenced to the internal residual proton or carbon resonances of $CDCl_3$ (^{1}H: 7.26 ppm, ^{13}C: 77.16 ppm) and C_4D_8O (^{1}H: 1.73 or 3.58 ppm, ^{13}C: 25.31 or 67.21 ppm). ^{2}H NMR chemical shifts are reported referenced to the internal deuterated solvent resonances of $CDCl_3$ at 7.26 ppm. Solution state magnetic susceptibility was measured by Evans' method[2] in THF with tetramethyl silane as an internal standard. Solid state magnetic susceptibility was measured by a magnetic susceptibility balance (Sherwood Scientific MSB-AUTO). Corrections were applied for diamagnetism calculated for Pascal constants.[3] Elemental analyses were performed on a Perkin Elmer 2400II and an Elementar VarioMicroCube. High-resolution mass spectra (HRMS) were measured by FAB (JEOL JMS-700 MStation FAB-MS) and ESI-TOF (Bruker micrOTOF II-SKA) mass spectrometers.

Synthesis of $CrBr_2$

Anhydrous $CrBr_2$ was prepared according to the previously reported procedure in *Inorganic Syntheses X* from chromium(0) with hydrobromic acid.[4] To a chromium(0) powder (60-100 μm, 5.88 g, 113 mmol) was added hydrobromic acid (47 wt%, 33 mL, 284 mmol) at 0 °C under an argon atmosphere. The reaction mixture was stirred for 3 hours at 0 °C, resulting in gradual formation of a blue solid along with a green solution and evolution of H_2. The blue solid, $[CrBr_2(H_2O)_6]$,[4] was collected by filtration on a glass frit under argon and washed with degassed acetone (10 mL x 3) until the Cr^{3+} green solution was removed completely. The blue solid on the glass frit was dissolved and extracted with degassed H_2O (40 °C, ca. 20 mL), and then filtered through the same glass frit to remove the unreacted chromium(0) powder. The collected blue solution was dried under vacuum to give a pale blue solid. The blue residue was further dried under vacuum at 100 °C and then above 150 °C until the solid changed in color to beige. The beige solid was collected in a vial inside a glove box to afford anhydrous $CrBr_2$ as a beige powder (12.8 g, 60.4 mmol, 53%). The purity of the obtained $CrBr_2$ was confirmed by UV-Vis measurement (Fig. S1) in degassed H_2O as $[Cr(H_2O)_6]^{2+}$ (λ_{max} = 716 nm, ε = 5.3 $M^{-1}\cdot cm^{-1}$), which was almost identical to a commercial $CrCl_2$ (99.99%, Sigma-Aldrich) in H_2O (λ_{max} = 717 nm, ε = 5.2 $M^{-1}\cdot cm^{-1}$).

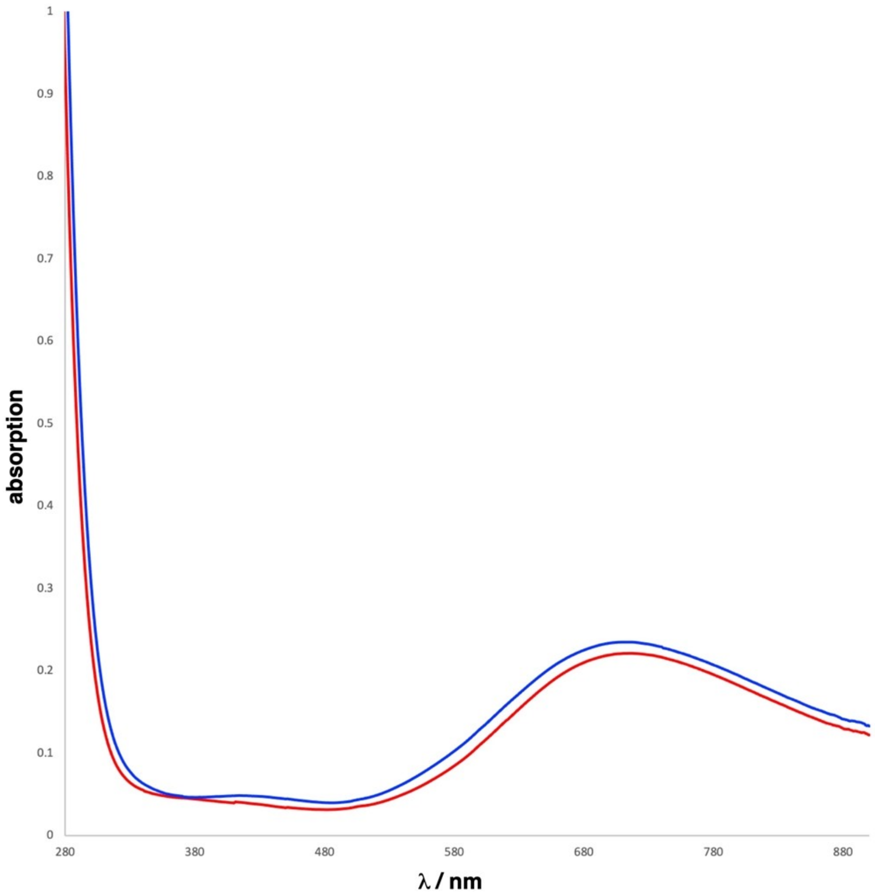

Fig. S1 UV-Vis spectra of $CrBr_2$ and commercial $CrCl_2$ in H_2O (blue: $CrBr_2$ in H_2O, 44.6 mM, red: commercial $CrCl_2$ in H_2O, 42.9 mM).

Synthesis of $[CrBr_2(thf)_2)][CrBr_2(thf)_3)](\mu\text{-}C)$ (4-thf)

To a pale blue suspension of $CrBr_2$ (1.50 g, 7.08 mmol) in THF (30 mL) was added a THF solution (3 mL) of carbon tetrabromide (340 mg, 1.03 mmol) at 0 °C, resulting in a rapid color change and formation of a mixture of a green solution and an orange solid, which was confirmed as $[CrBr_3(thf)_3]$[5] by X-ray crystallography. The reaction mixture was stirred for 30 minutes at 0 °C. The orange solid was removed by centrifugation and the green supernatant was collected. All volatile materials of the obtained green solution was removed quickly under vacuum. The greenish brown residue was washed with a mixture of hexane and THF (5 x 5 mL, hexane : THF = 3 : 1) until the supernatant changed from orange to slightly green. The green solid was dried under vacuum to yield **1** (728 mg, 914 μmol, 90% yield). Green crystals of **4-thf** were grown from a concentrated THF solution layered with pentane at −20 °C. Complex **4-thf** is stable in solid-state at room temperature, but unstable in solution even at low temperature (–78 °C). Upon crystallization of **4-thf** from a THF solution at room temperature, reddish brown crystals were obtained along with formation of $[CrBr_3(thf)_3]$. IR (cm^{-1}; KBr): 580, 684, 787, 862, 920, 956, 1012, 1040, 1143, 1180, 1244, 1313, 1341, 1361, 1448, 1457, 1489 2878, 2903, 2982. μ_{eff} (Evans' Method, THF, 25 °C): 4.84 μ_B. μ_{eff} (Solid, 24.8 °C): 5.03 μ_B. UV-Vis (THF, λ_{max}/nm (ε/$M^{-1}\cdot cm^{-1}$)): 647 (303). Anal. Calcd. for $C_{21}H_{40}O_5Br_4Cr_2$: C, 31.68; H, 5.06. Found: C, 32.02; H, 4.76.

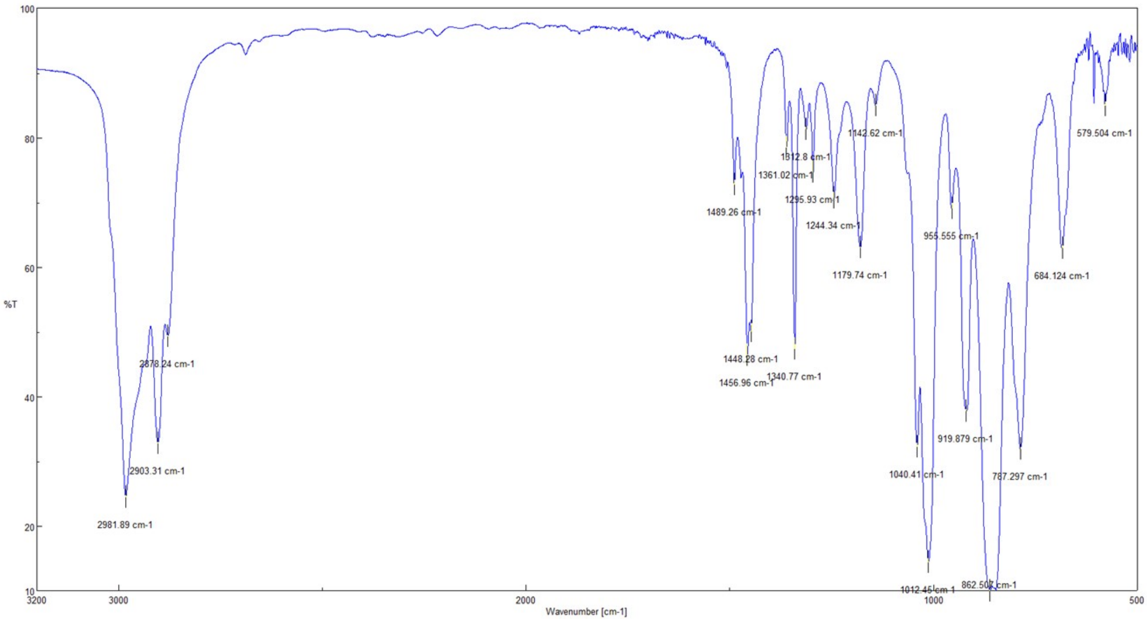

Fig. S2 IR spectrum of **4-thf** (KBr, solid, room temperature).

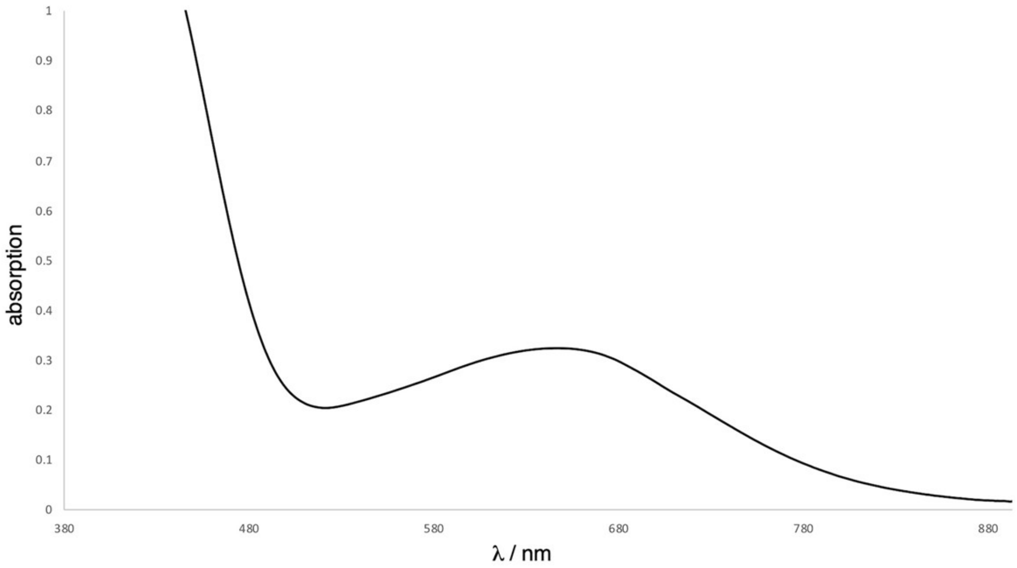

Fig. S3 UV-Vis spectrum of **4-thf** (THF, room temperature).

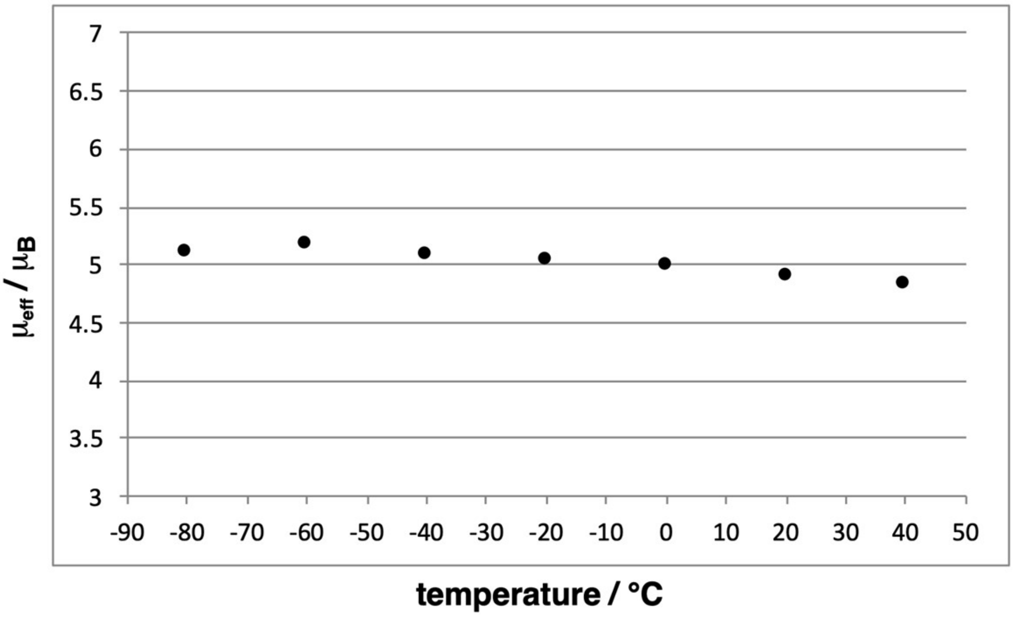

Fig. S4 VT-Evans' method of **4-thf** from –80 °C to +40 °C.

Synthesis of [$CrBr_2$(thf)$_2$)][$CrBr_2$(thf)$_3$)](μ-^{13}C) (4-^{13}C)

To a pale blue suspension of $CrBr_2$ (698 mg, 3.30 mmol) in THF (15 mL) was added a THF solution (5 mL) of $^{13}CBr_4$ (150 mg, 451 μmol) at 0 °C, resulting in a rapid color change and formation of a mixture of a green solution and an orange solid. The reaction mixture was stirred for 30 minutes at 0 °C. The orange solid was removed by centrifugation and the green supernatant was collected. All volatile materials of the obtained green solution was removed quickly under vacuum. The greenish brown residue was washed with a mixture of hexane and THF (3 x 3 mL, hexane : THF = 3 : 1) until the supernatant changed from orange to slightly green. The green solid was dried under vacuum to yield **4-^{13}C** (340 mg, 427 μmol, 95% yield). The ^{13}C{^{1}H} NMR spectrum of **4-^{13}C** was measured in THF-d_8, but none of ^{13}C NMR signals other than solvents could be observed from –2000 ppm to +2000 ppm. Further measurement of **4-^{13}C** in THF-d_8 resulted in decomposition of the NMR sample due to instability of the chromium carbide in solution.

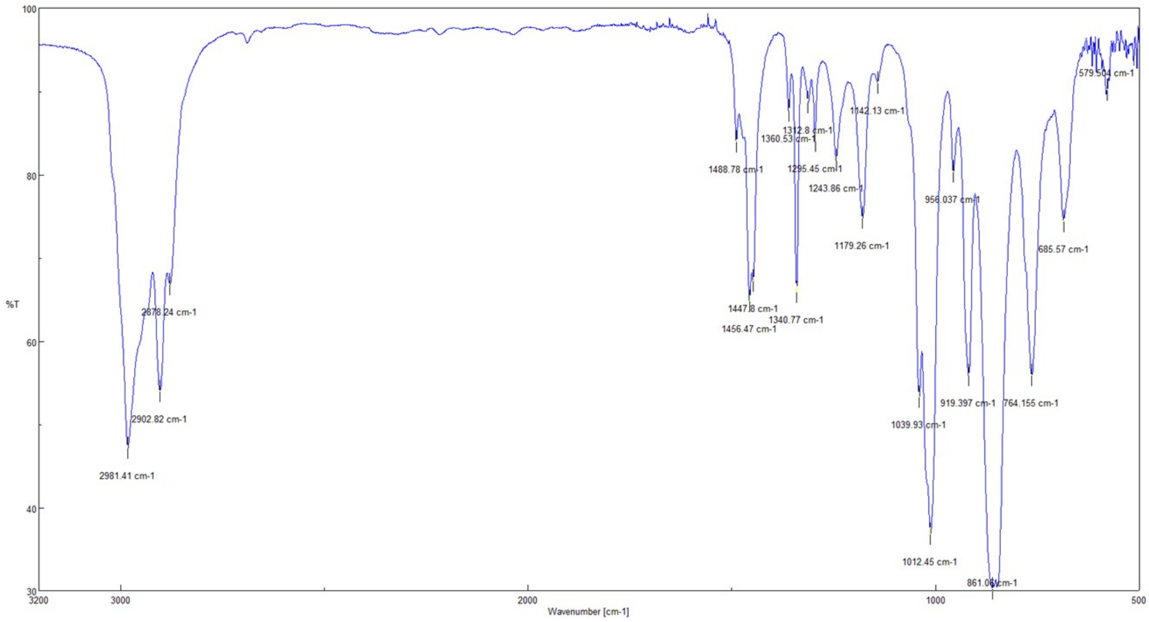

Fig. S5 IR spectrum of **4-^{13}C** (KBr, solid, room temperature).

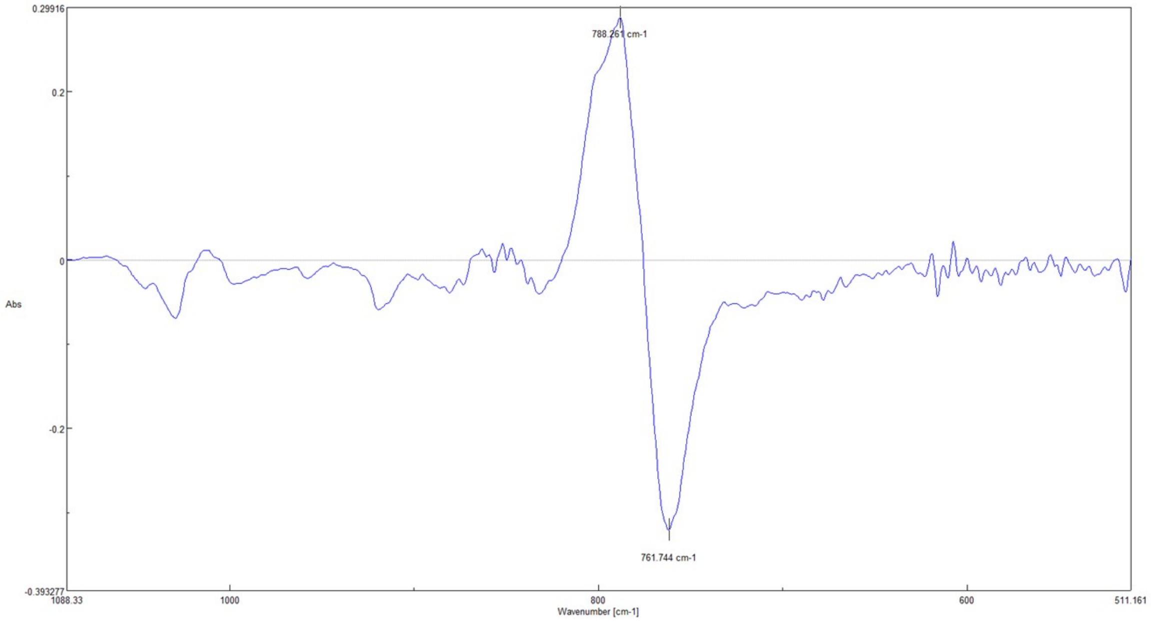

Fig. S6 Difference IR spectrum of **4**/**4-^{13}C** (positive: **4**, negative: **4-^{13}C**).

Reaction of 3-phenylpropanal with $CrBr_2$ and CBr_4

i) Method A

To a pale blue suspension of $CrBr_2$ (666 mg, 3.14 mmol) in THF (10 mL) was added 3-phenylpropanal (138 mg, 1.03 mmol) dropwise at 0 °C. To a mixture solution was added a THF solution (3 mL) of carbon tetrabromide (165 mg, 498 μmol) dropwise at 0 °C. The reaction mixture was stirred for 15 minutes at 0 °C, then warmed up to room temperature and stirred additionally for 24 hours, resulting in a gradual color change to brown. The reaction mixture was quenched by H_2O (ca. 20 mL) under air. The yields of **1a-Br**, **2a**, and **3a** based on carbon tetrabromide were determined by FID-GC analyses with mesitylene as an internal standard (**1a-Br**: 307 μmol, 62% yield; **2a**: 69.2 μmol, 14% yield, **3a**: 7.7 μmol, 2% yield).

ii) Method B

To a pale blue suspension of $CrBr_2$ (662 mg, 3.13 mmol) in THF (10 mL) was added a THF solution (3 mL) of carbon tetrabromide (164 mg, 495 μmol) dropwise at 0 °C, resulting in a rapid color change and formation of a mixture of a green solution and an orange solid. The reaction mixture was stirred for 1 hour at 0 °C. To the green solution was added 3-phenylpropanal (134 mg, 999 μmol) dropwise at 0 °C, warmed up to room temperature and stirred additionally for 24 hours, resulting in a gradual color change to brown. The reaction mixture was quenched by H_2O (ca. 20 mL) under air. The yields of **1a-Br**, **2a**, and **3a** based on carbon tetrabromide were determined by FID-GC analyses with mesitylene as an internal standard (**1a-Br**: 29.1 μmol, 6% yield; **2a**: 44.2 μmol, 9% yield, **3a**: 129 μmol, 26% yield).

Further characterization and isolation of **1a-Br**, **2a**, and **3a** were performed by work-up of the extracted organic phase with Et_2O (20 mL x 3). The combined organic phase was washed with brine, dried over magnesium sulfate, filtered and evaporated by a rotary evaporator to give a pale green oil. Compound **1a-Br**, **2a**, and **3a** were separated from the crude product by column chromatography with a mixture of hexane and ethyl acetate in a 5:1 ratio, **1a-Br** (R_f = 0.29, hexane : ethyl acetate = 5:1),

2a (R_f = 0.58, hexane : ethyl acetate = 5:1), and **3a** (R_f = 0.43, hexane : ethyl acetate = 5:1). Purities of **2a** and **3a** were confirmed by comparison to the ^{1}H NMR spectra of a commercially available sample for **2a** and the reported spectrum in the previous paper for **3a**.[6]

1a-Br: ^{1}H NMR (400 MHz, chloroform-*d*, 25 °C): δ 7.32-7.26 (m, overlapped with $CHCl_3$, 4H, Ph), 7.22-7.15 (m, 6H, Ph), 6.00 (t, J_{HH} = 7 Hz, 1H, CH_2CH=CBr), 4.05 (q, J_{HH} = 6 Hz, 1H, C*H*OH), 2.77-2.73 (m, 2H, CH_2CH=CBr), 2.61-2.53 (m, 4H, CH_2Ph), 1.97 (q, J_{HH} = 7 Hz, 2H, CH_2CHOH), 1.82 (d, J_{HH} = 6 Hz, 1H, CHO*H*). ^{13}C{^{1}H} NMR (101 MHz, chloroform-*d*, 25 °C) δ 141.48 (Ph), 141.15 (Ph), 132.04 (CH_2CH=*C*Br), 129.90 (CH_2*C*H=CBr), 128.58 (Ph), 128.56 (Ph), 126.28 (Ph), 126.10 (Ph), 76.01 (CH_2*C*HOH), 37.74 (*C*H_2CHOH), 34.45 (*C*H_2CH=CBr), 32.47 (*C*H_2Ph), 31.64 (*C*H_2Ph). HRMS (FAB$^+$) Calcd for $C_{19}H_{21}OBr$ $[M]^+$: 344.0776; Found: 344.0786.

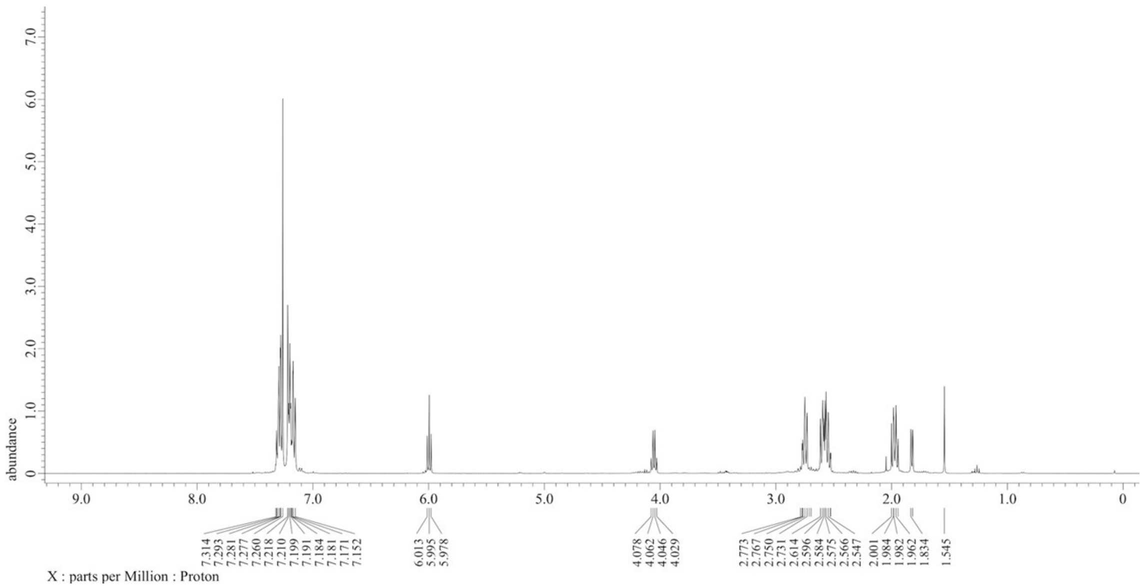

Fig. S7 ^{1}H NMR spectrum of **1a-Br** (400 MHz, in chloroform-*d*, at 25 °C).

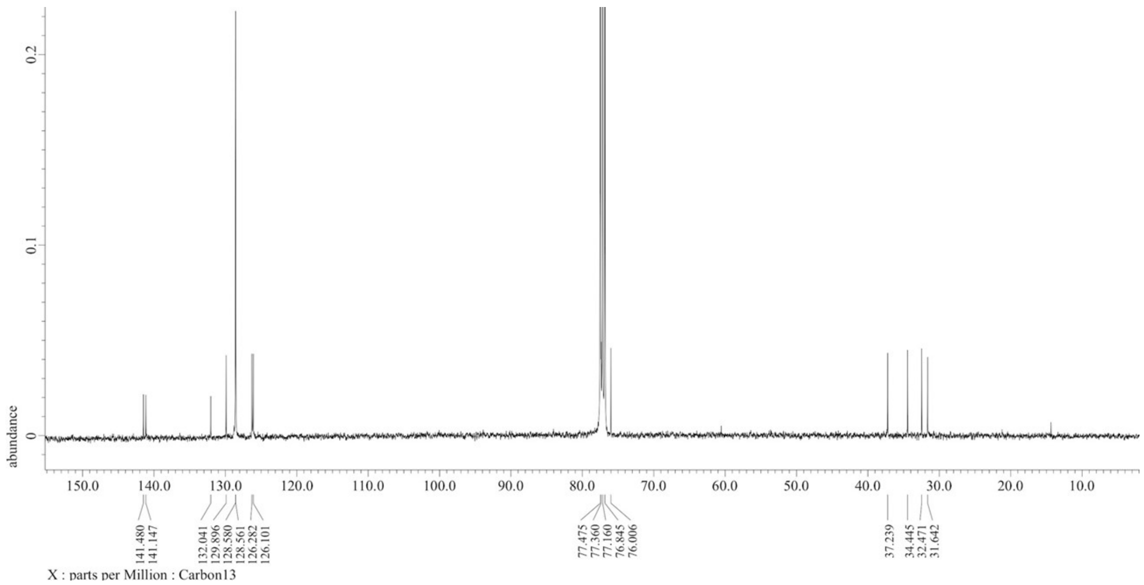

Fig. S8 ^{13}C{^{1}H} NMR spectrum of **1a-Br** (101 MHz, in chloroform-*d*, at 25 °C).

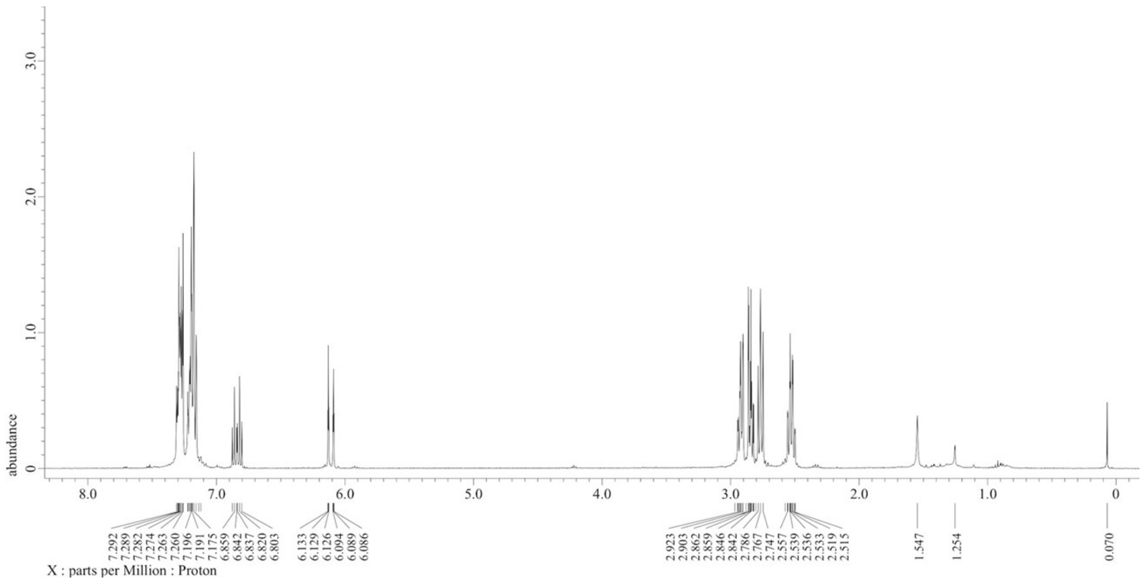

Fig. S9 ^{1}H NMR spectrum of **3a** (400 MHz, in chloroform-*d*, at 25 °C).

Reaction of 3-Phenylpropanal with 4-thf

To a green solution of **4-thf** (388 mg, 487 μmol) in THF (15 mL) was added 3-phenylpropanal (134 mg, 1.00 mmol) dropwise at 0 °C. The reaction mixture was warmed up to room temperature and stirred additionally for 2 hours, resulting in a gradual color change to orange. The reaction mixture was quenched by H_2O (ca. 10 mL) under air. The yield of **3a** was determined by FID-GC analyses with mesitylene as an internal standard (**3a**: 105 μmol, 22% yield). Analyses of the crude product by ^{1}H NMR and GC-MS revealed a complicated mixture delivered from **3a** by activation of THF. Formation of these side-products might be the reason why the yield of the resulted α,β-unsaturated ketone **3a** was low. Attempts to isolate **3a** from this mixture by column chromatography lowered the isolated yield of **3a** (20.4 μmol, 4% yield).

Reaction of Benzaldehyde with 4-thf

To a green solution of **4-thf** (374 mg, 470 μmol) in THF (15 mL) was added benzaldehyde (105 mg, 984 μmol) dropwise at 0 °C. The reaction mixture was warmed up to room temperature and stirred additionally for 2 hours, resulting in a gradual color change to orange. The reaction mixture was quenched by H_2O (ca. 10 mL) under air. The yield of **3b** was determined by FID-GC analyses with mesitylene

as an internal standard (**3b**: 95.8 μmol, 20% yield). Further characterization and isolation of **3b** were performed by work-up of the extracted organic phase with Et_2O (20 mL x 3). The combined organic phase was washed with brine, dried over magnesium sulfate, filtered and evaporated by a rotary evaporator to give a pale green oil. Compound **3b** were separated from the crude product by column chromatography with a mixture of hexane and ethyl acetate in a 5:1 ratio, **3b** (R_f = 0.52, hexane : ethyl acetate = 5:1). Purity of **3b** was confirmed by comparison to the ^{1}H NMR spectrum of a commercially available sample. Akin to **3a**, analyses of the crude product by ^{1}H NMR and GC-MS spectroscopy revealed a complicated mixture delivered from **3b** by activation of THF and the isolated yield of **3b** was low (24.2 μmol, 5%).

Reaction of 3-Phenylpropanal with 4-^{13}C

To a green solution of **4-^{13}C** (370 mg, 464 μmol) in THF (15 mL) was added 3-phenylpropanal (138 mg, 1.03 mmol) dropwise at 0 °C. The reaction mixture was stirred at 0 °C for 2 hours. The reaction mixture was quenched by H_2O (ca. 10 mL) under argon and then exposed to air. The organic phase was extracted with Et_2O (20 mL x 3). The combined organic phase was dried over magnesium sulfate and filtered. The filtrate was evaporated by a rotary evaporator to give a yellowish green oil. The crude product was by a eluent, hexane : ethyl acetate = 10:1, to yield **3a-^{13}C** (18.0 mg, 68.1 μmol, 15% crude yield), but separation from some ^{13}C-enriched side products failed. The ^{1}H NMR spectrum of **3a-^{13}C** (Fig. S10) showed a doublet of doublets of triplets at 6.10 ppm ($^1J_{CH}$ = 157 Hz) and the ^{13}C{^{1}H} NMR spectrum of **3a-^{13}C** showed an enriched-^{13}C signal at 130.85 ppm (Fig. S11), which showed a strong cross-peak with ^{1}H: 6.10 ppm by ^{1}H-^{13}C HMQC (Fig. S12). GC-MS analysis of **3a-^{13}C** (Fig. S33) showed m/z = 265, instead of m/z = 264 (**3a**)

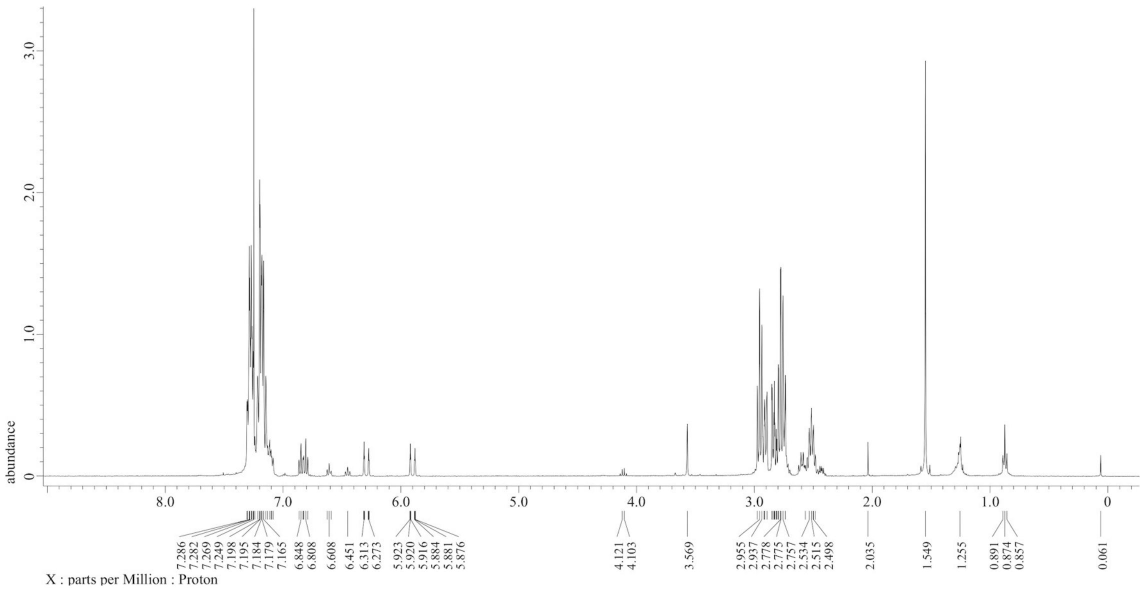

Fig. S10 ^{1}H NMR spectrum of **3a-^{13}C** (400 MHz, in chloroform-*d*, at 25 °C).

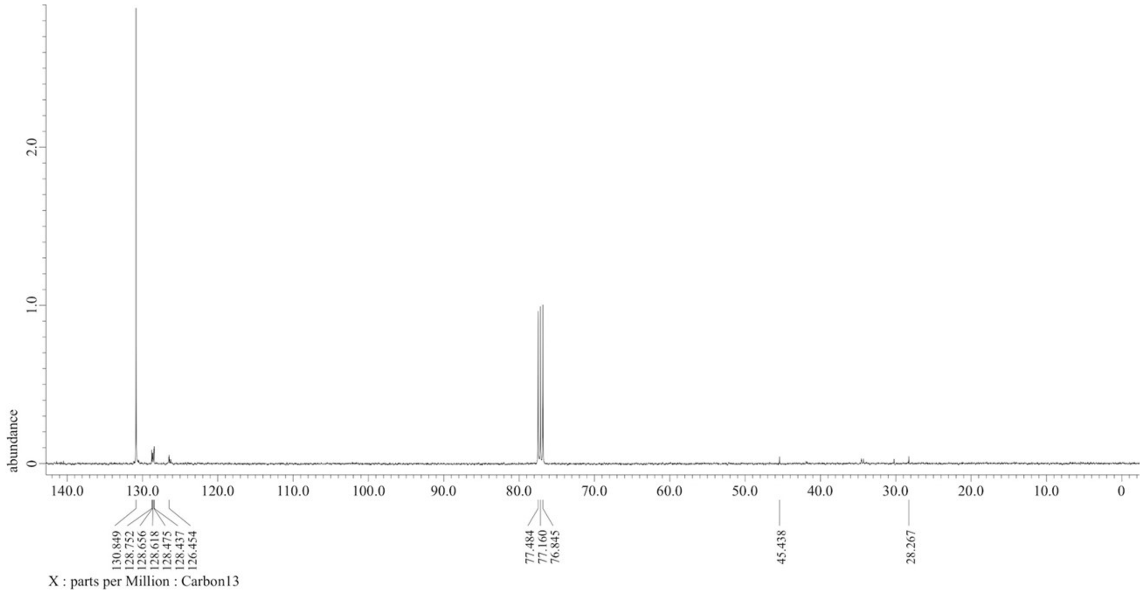

Fig. S11 ^{13}C{^{1}H} NMR spectrum of **3a-^{13}C** (101 MHz, in chloroform-*d*, at 25 °C).

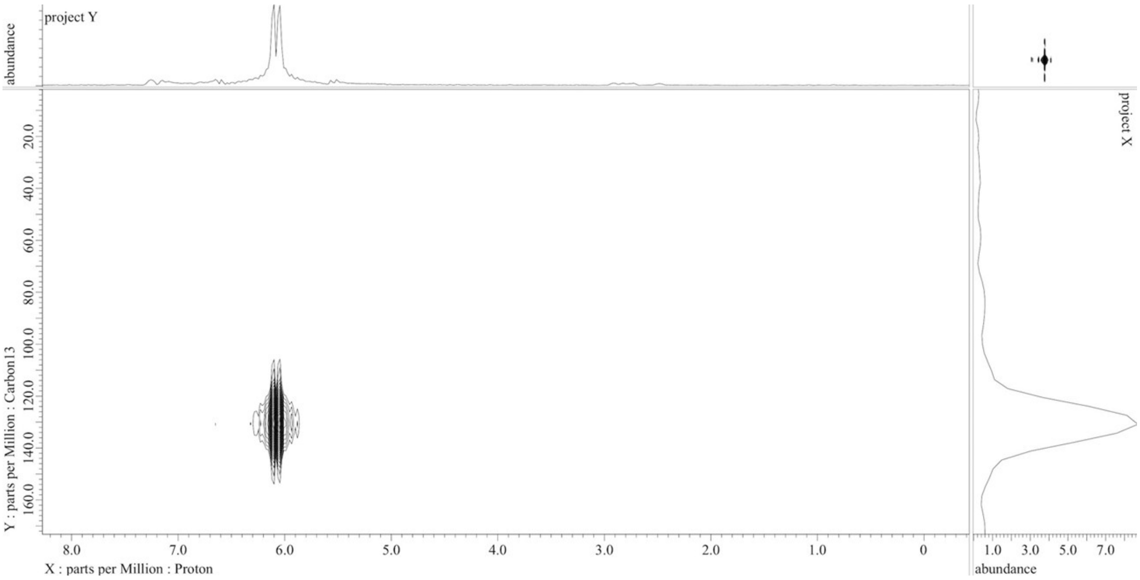

Fig. S12 ^{1}H-^{13}C HMQC NMR spectrum of **3a-^{13}C** (400 MHz, in dichloromethane-d_2, at 25 °C).

Reaction of 3-Phenylpropanal-*d* with 4-thf

To a green solution of **4-thf** (800 mg, 1.00 mmol) in THF (20 mL) was added 3-phenylpropanal-*d* (293 mg, 2.17 mmol) dropwise at 0 °C. The reaction mixture was stirred at 0 °C for 2 hours. The reaction mixture was quenched by H_2O (ca. 10 mL) under argon and then exposed to air. The organic phase was extracted with Et_2O (20 mL x 3). The combined organic phase was dried over magnesium sulfate and filtered. The filtrate was evaporated by a rotary evaporator to give a pale yellowish greenish oil. The crude product was by a eluent, hexane : ethyl acetate = 10:1, to yield **3a-*d*** (24 mg, 91.2 μmol, 9%). The ^{1}H NMR spectrum of **3a-*d*** showed no signals around 6.1 ppm and 6.8 ppm corresponding to the α- and β-protons of **3a**, while the ^{2}H NMR spectrum showed two signals at 6.15 ppm and 6.88 ppm. GM-MS analysis of **3a-*d*** (Fig. S33) showed m/z = 266, instead of m/z = 264 (**3a**).

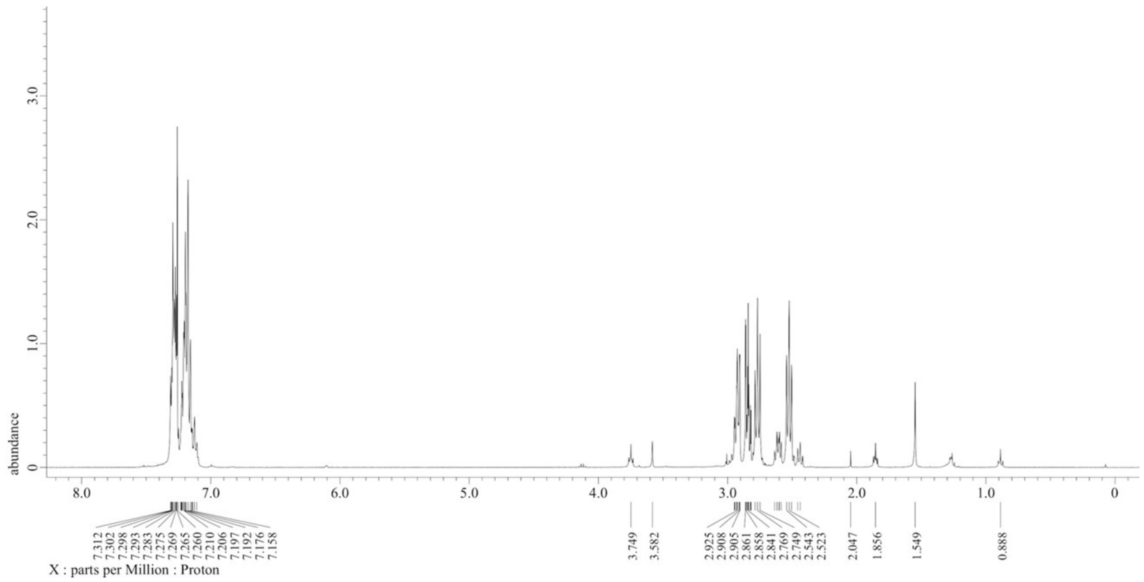

Fig. S13 ^{1}H NMR spectrum of **3a-*d*** (400 MHz, in chloroform-*d*, at 25 °C).

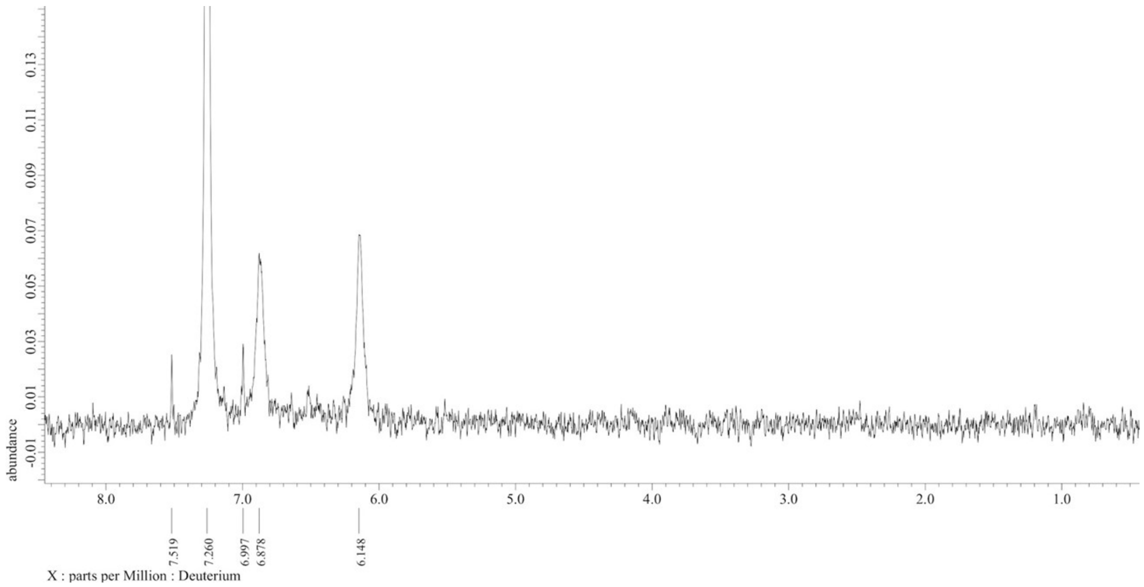

Fig. S14 ^{2}H NMR spectrum of **3a-*d*** (62 MHz, in chloroform/chloroform-*d*, at 25 °C).

Reaction of 4-Phenyl-1-butene with 4-thf

i) Method A

To a green solution of **4-thf** (809 mg, 1.02 mmol) in THF (10 mL) was added 4-phenyl-1-butene (268 mg, 2.03 mmol) dropwise at 0 °C. The reaction mixture was warmed up to room temperature and stirred additionally for 24 hours, resulting in a gradual color change to clear yellow and formation of orange solid. The reaction mixture was quenched by H_2O (ca. 10 mL) under air. The organic phase was extracted with Et_2O (10 mL x 3). The combined organic phase was dried over magnesium sulfate, filtered and evaporated by a rotary evaporator to give a pale yellow oil. The crude product was purified by column chromatography with a mixture of hexane and ethyl acetate in a 10:1 ratio (R_f = 0.23, hexane : ethyl acetate = 50:1) to give a mixture of diastereomers of **6a** (67.3 mg, 243 μmol, 24% yield based on **4-thf**, *distal* : *proximal* : *medial* = 28:17:55).

ii) Method B

To a pale blue suspension of $CrBr_2$ (1.29 g, 6.09 mmol) in THF (10 mL) was added a THF solution (5 mL) of carbon tetrabromide (337 mg, 1.02 mmol) dropwise at 0 °C, resulting in a rapid color change and formation of a mixture of a green solution and an orange solid. The reaction mixture was stirred for 30 minutes at 0 °C. To the green solution was added 4-phenyl-1-butene (273 mg, 2.07 mmol) dropwise at 0 °C, warmed up to room temperature and stirred additionally for 24 hours, resulting in a gradual color change to brown. The reaction mixture was quenched by H_2O (ca. 20 mL) under air. The yield of **6a** as a mixture of diastereomers[7] based on carbon tetrabromide was determined by FID-GC analyses with mesitylene as an internal standard (**3a**: 226 μmol, 22% yield based on CBr_4, *distal* : *proximal* : *medial* = 32:20:48).

6a (mixture of diastereomers): ^{1}H NMR (400 MHz, chloroform-*d*, 25 °C): δ 7.27 (t, J_{HH} = 8 Hz, 4H, Ph), 7.19 (d, J_{HH} = 8 Hz, 4H, Ph), 7.18 (t, J_{HH} = 8 Hz, 2H, Ph), 2.80-2.58 (m, overlapped, CH_2CH_2Ph, *distal* 4H + *proximal* 2H + *medial* 4H), 2.02 (m, 2H,

CH_2CH_2Ph, *proximal*), 1.65-1.56 (m, overlapped, C*H*$_2$CH$_2$Ph, *distal* 4H + *medial* 4H), 1.30 (m, 4H, C*H*$_2$CH$_2$Ph, *proximal*), 1.18 (m, 2H, spiro-CH, *proximal*), 1.08 (m, 1H, spiro-CH, *medial*), 1.00 (m, 2H, spiro-CH, *distal*), 0.91 (m, 1H, spiro-CH, *medial*), 0.85 (dd, J_{HH} = 4.0 Hz, 8 Hz, spiro-CH$_2$, *proximal* 2H + *medial* 1H), 0.80 (dd, J_{HH} = 4.0 Hz, 8 Hz, 1H, spiro-CH$_2$, *medial*), 0.74 (dd, J_{HH} = 4.0 Hz, 8 Hz, 2H, spiro-CH$_2$, *distal*), 0.42-0.40 (overlapped t, J_{HH} = 4.0 Hz, 2H, spiro-CH$_2$, *proximal* 2H + *medial* 2H) 0.27 (t, J_{HH} = 4.0 Hz, 2H, spiro-CH$_2$, *distal*). $^{13}C\{^1H\}$ NMR (101 MHz, chloroform-*d*, 25 °C) δ 142.86 (Ph), 142.81 (Ph), 142.79 (Ph), 128.62 (Ph), 128.55 (Ph), 128.38 (Ph), 128.34 (Ph), 125.76 (Ph), 36.39 (CH$_2$*C*H$_2$Ph), 35.90 (CH$_2$*C*H$_2$Ph), 35.86 (CH$_2$*C*H$_2$Ph), 35.34 (*C*H$_2$CH$_2$Ph), 35.22 (*C*H$_2$CH$_2$Ph), 34.96 (*C*H$_2$CH$_2$Ph), 34.73 (*C*H$_2$CH$_2$Ph), 21.22 (spiro-C, *distal*), 21.19 (spiro-C, *medial*), 19.51 (spiro-C, *proximal*), 18.54 (spiro-CH, *proximal*), 17.78 (spiro-CH, *medial*), 17.53 (spiro-CH, *distal*), 15.00 (spiro-CH, *medial*), 12.79 (spiro-CH$_2$, *proximal*), 9.98 (spiro-CH$_2$, *medial*), 9.92 (spiro-CH$_2$, *distal*). HRMS (ESI$^+$) Calcd for $C_{21}H_{24}$ [M]$^+$: 276.1878; [M+Na]$^+$: 299.1776, Found: 299.1784.

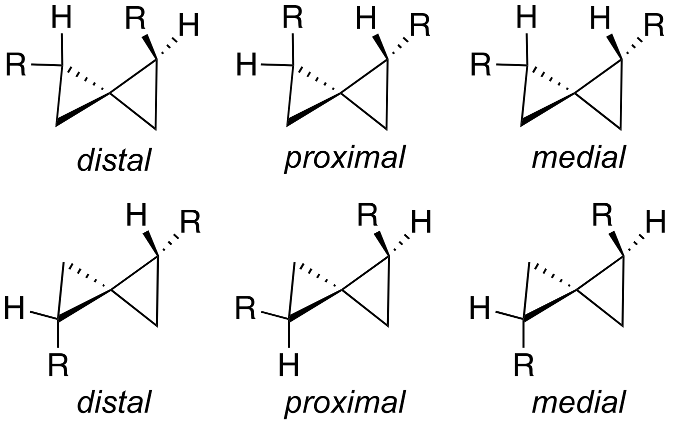

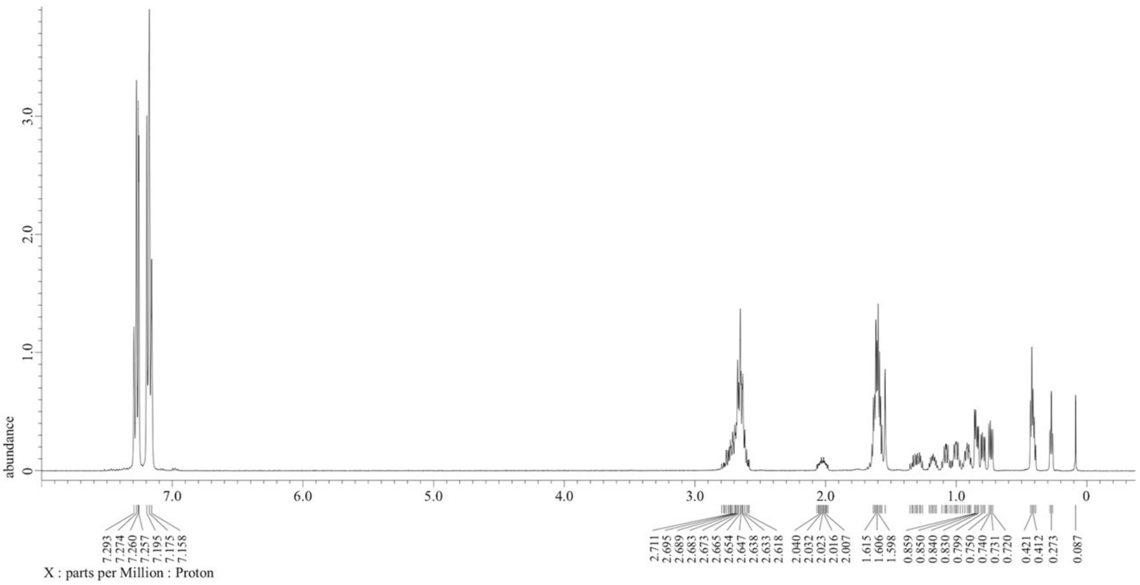

Fig. S15 ^{1}H NMR spectrum of **6a** by Method B (400 MHz, in chloroform-*d*, at 25 °C).

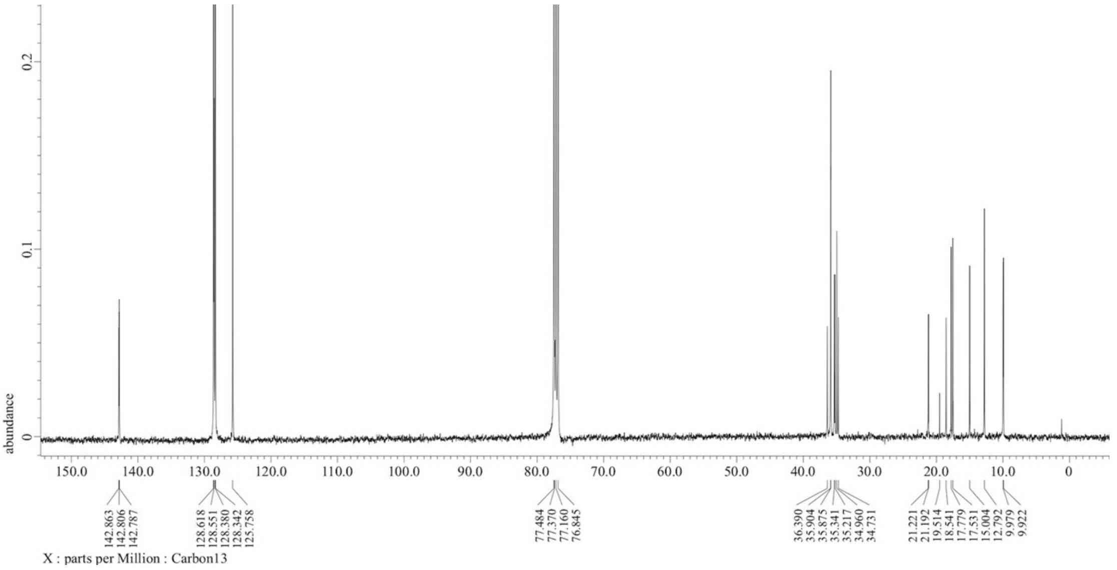

Fig. S16 ^{13}C{^{1}H} NMR spectrum of **6a** by Method B (101 MHz, in chloroform-*d*, at 25 °C).

Reaction of 4-phenyl-1-butene with 4-^{13}C

To a green solution of **4-^{13}C** (403 mg, 506 μmol) in THF (5 mL) was added 4-phenyl-1-butene (140 mg, 1.06 mmol) dropwise at 0 °C. The reaction mixture was warmed up to room temperature and stirred additionally for 1 hour, resulting in a gradual color change to clear yellow and formation of orange solid. The reaction mixture was quenched by H_2O (ca. 5 mL) under air. The organic phase was extracted with Et_2O (10 mL x 3). The combined organic phase was dried over magnesium sulfate, filtered and evaporated by a rotary evaporator to give a pale yellowish oil. The crude product was purified by column chromatography with a mixture of hexane and ethyl acetate (R_f = 0.23, hexane : ethyl acetate = 50 : 1) to give a mixture of diastereomers of **6a-^{13}C** (25.0 mg, 94 μmol, 19% yield based on **4-^{13}C**). The ^{13}C{^{1}H} NMR spectrum of **6a-^{13}C** showed enriched ^{13}C NMR signals at 19.51 ppm, 21.19 ppm and 21.22 ppm (Fig. S18).

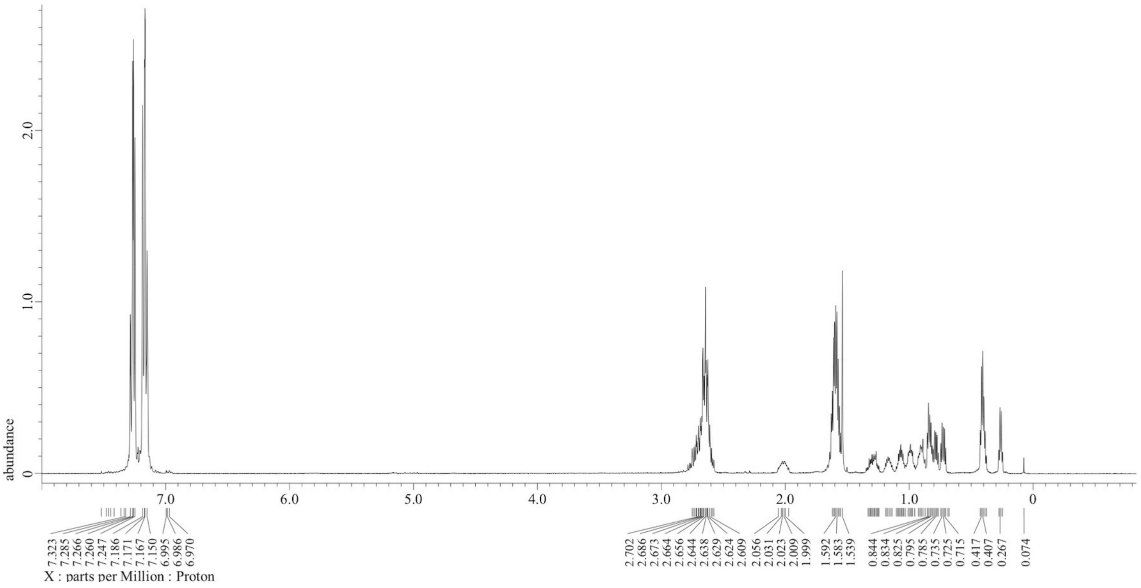

Fig. S17 ^{1}H NMR spectrum of **6a-^{13}C** (400 MHz, in chloroform-*d*, at 25 °C).

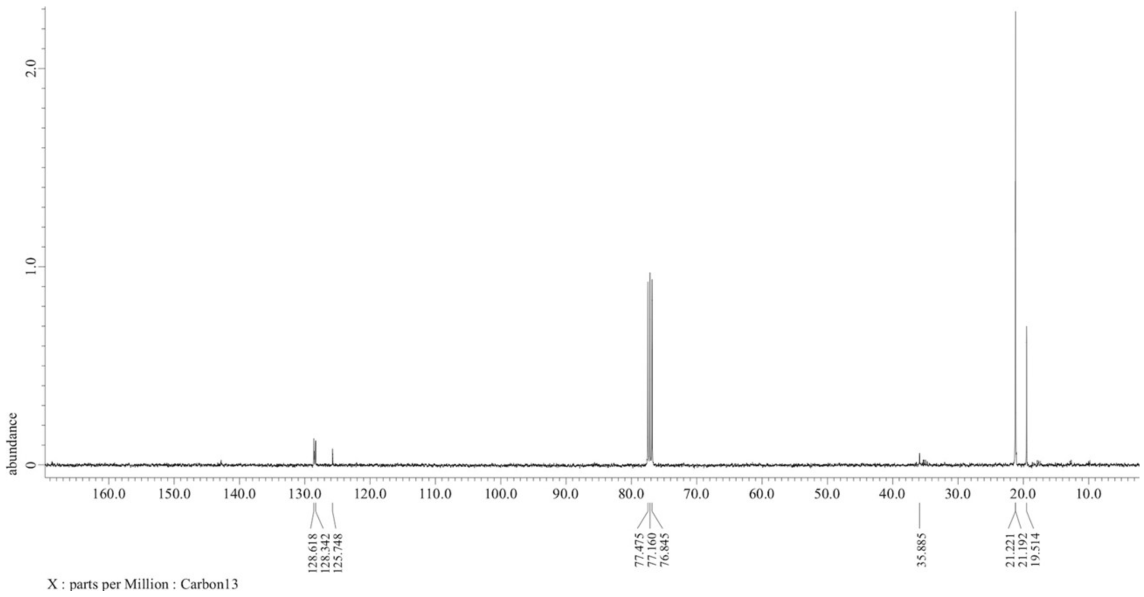

Fig. S18 $^{13}C\{^1H\}$ NMR spectrum of **6a-^{13}C** (101 MHz, in chloroform-*d*, at 25 °C).

Reaction of ethylene with 4-thf

In a 30 mL Schlenk tube, **4-thf** (47.0 mg, 59.0 μmol) was dissolved in THF-d_8 (ca. 1 mL). The headspace was evacuated, and an atmosphere of ethylene (1 atm) was introduced. The mixture was stirred at room temperature for 1 hour, resulting in a gradual color change to clear yellow and formation orange solid. All volatile materials were transferred under vacuum into a J-Young valve NMR tube containing biphenyl as an internal standard. The ^{1}H NMR spectrum of the transferred sample showed only **6b** (9.4 μmol, 16% yield), THF, ethylene, and biphenyl (Fig. S19).

6b: ^{1}H NMR (400 MHz, THF-d_8, 25 °C): δ 0.74 (s, 4H, CH_2). $^{13}C\{^1H\}$ NMR (101 MHz, THF-d_8, 25 °C): δ 9.29 (spiro-C), 6.51 (spiro-CH_2).

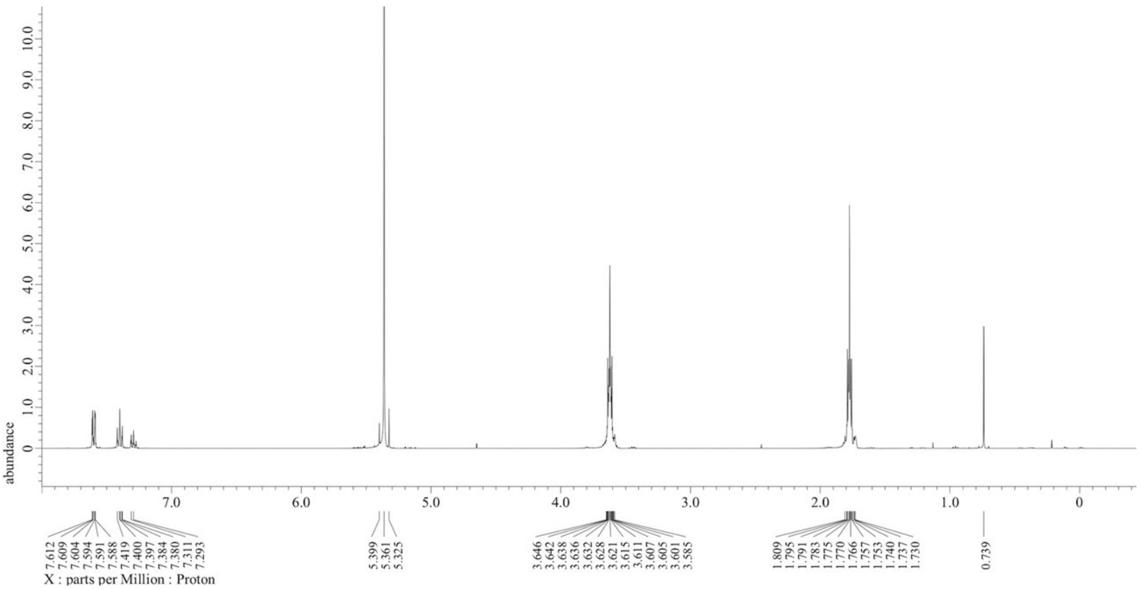

Fig. S19 ^{1}H NMR spectrum of **6b** with ethylene and biphenyl (400 MHz, in THF-d_8, at 25 °C).

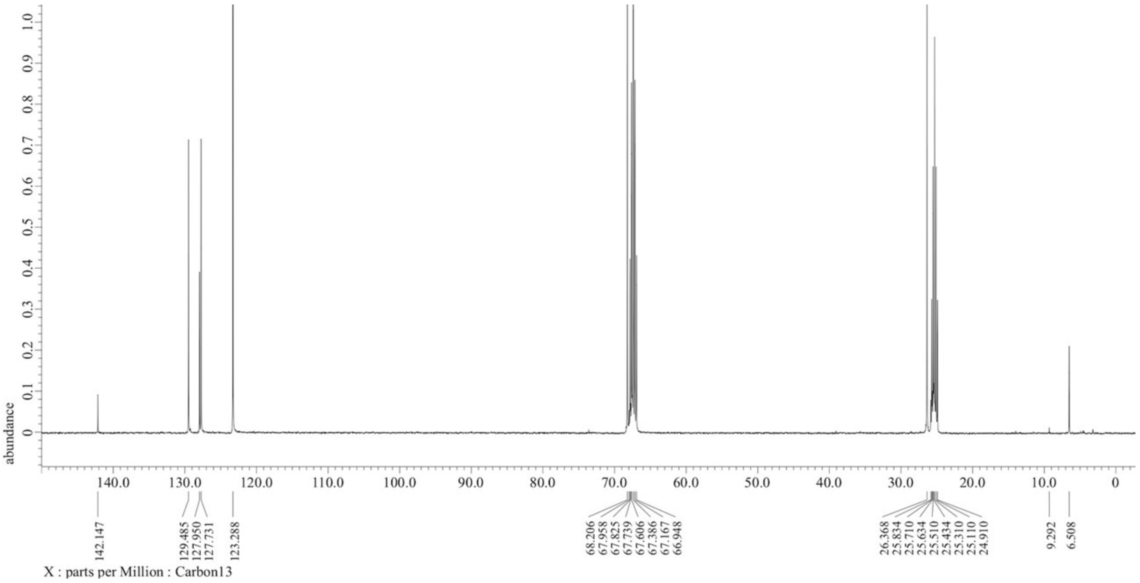

Fig. S20 ^{13}C{^{1}H} NMR spectrum of **6b** with ethylene and biphenyl (101 MHz, in THF-d_8, at 25 °C).

Reaction of ethylene with 4-^{13}C

In a 30 mL Schlenk tube, **4-^{13}C** (63.1 mg, 79.2 μmol) was dissolved in THF-d_8 (ca. 1 mL). The headspace was evacuated, and an atmosphere of ethylene (1 atm) was introduced. The mixture was stirred at room temperature for 1 hour, resulting in a gradual color change to clear yellow and formation orange solid. All volatile materials were transferred under vacuum into a J-Young valve NMR tube containing biphenyl as an internal standard. The ^{1}H NMR spectrum of the transferred sample showed **6b-^{13}C** (10.3 μmol, 13% yield) as a doublet at 0.74 ppm ($^2J_{CH}$ = 4 Hz). The ^{13}C{^{1}H} NMR spectrum of **6b-^{13}C** (Fig. S22) showed an enriched ^{13}C NMR signal at 9.30 ppm along with a doublet ($^1J_{CC}$ = 21 Hz) at 6.50 ppm.

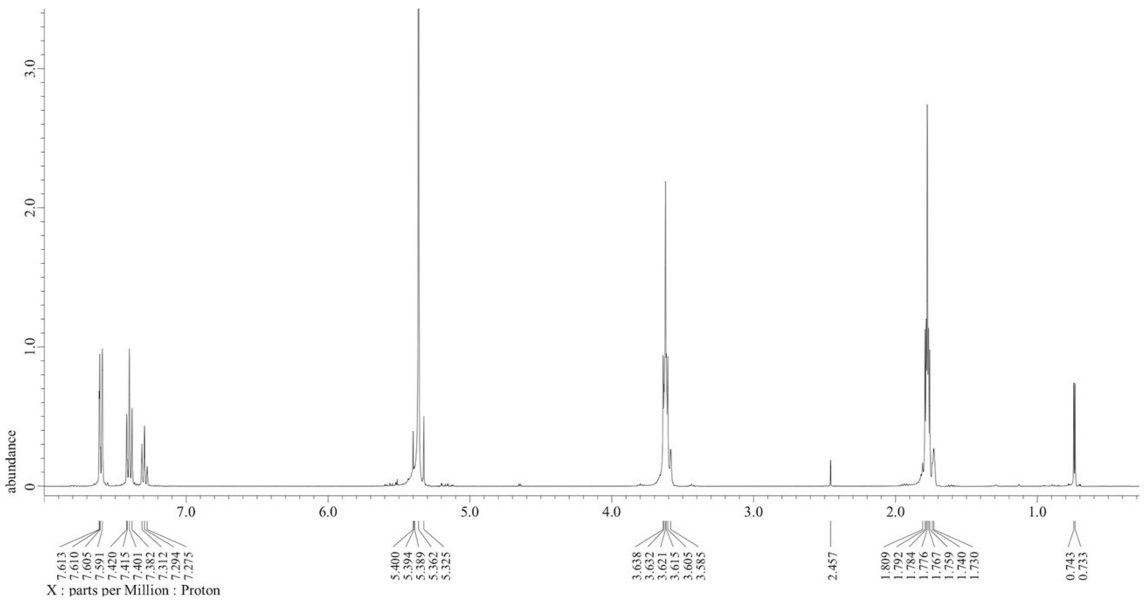

Fig. S21 ^{1}H NMR spectrum of **6b-^{13}C** with ethylene and biphenyl (400 MHz, in THF-d_8, at 25 °C).

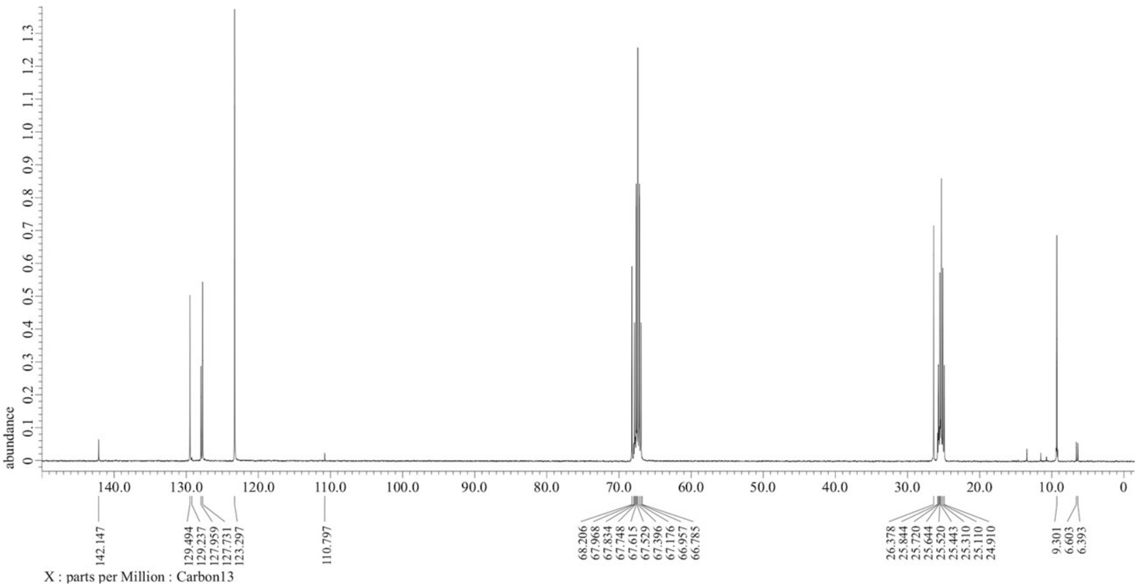

Fig. S22 $^{13}C\{^1H\}$ NMR spectrum of **6b-^{13}C** with ethylene and biphenyl (101 MHz, in THF-d_8, at 25 °C).

Synthesis of 7a

i) One equivalent of 4-thf

To a green solution of **4-thf** (380 mg, 477 μmol) in THF (10 mL) was added 4-phenyl-1-butyne (62.4 mg, 479 μmol) dropwise at 0 °C. The reaction mixture was warmed up to room temperature and stirred additionally for 1 hour, resulting in a gradual color change to light green along with formation of a white solid, which was confirmed as $[CrBr_2(thf)_2]_n$ by X-ray crystallography.[8] The white solid was removed by centrifugation and the green supernatant was collected. All volatile materials of the obtained green solution was removed under vacuum. The green residue was dissolved in DME (5 mL) as a green solution, while the remaining white solid $[CrBr_2(thf)_2]_n$ changed in color to purple. After filtration to remove the purple solid, the green solution was layered with hexane (10 mL) at –20 °C, resulting in formation of a green solid. The green solid was dried under vacuum to yield **7a** (71.2 mg, 136 μmol, 29% yield based on **4-thf**).

ii) Two equivalents of 4-thf

To a green solution of **4-thf** (820 mg, 1.03 mmol) in THF (10 mL) was added 4-

phenyl-1-butyne (64.5 mg, 495 μmol) dropwise at 0 °C. The reaction mixture was warmed up to room temperature and stirred additionally for 1 hour, resulting in a gradual color change to light green along with formation of a white solid. The white solid was removed by centrifugation and the green supernatant was collected. All volatile materials of the obtained green solution was removed under vacuum. The green residue was dissolved in DME (5 mL), while the remaining white solid $[CrBr_2(thf)_2]_n$ changed in color to purple. After filtration to remove the purple solid, the green solution was layered with hexane (10 mL) at –20 °C, resulting in formation of a green solid. The green solid was dried under vacuum to yield **7a** (115 mg, 219 μmol, 44% yield based on 4-phenyl-1-butyne).

7a: IR (cm^{-1}; KBr): 539, 700, 750, 759, 822, 827, 832, 842, 979, 1032, 1072, 1107, 1134, 1187, 1238, 1280, 1327, 1435, 1454, 1495, 1602, 1721, 2839, 2941, 3070. μ_{eff} (Evans' Method, DME, 20 °C): 3.92 μ_B. Anal. Calcd. for $C_{15}H_{20}O_2Br_3Cr$: C, 34.38; H, 3.85. Found: C, 33.79; H, 3.11. Multiple attempts to obtain satisfactory elemental analysis data failed.

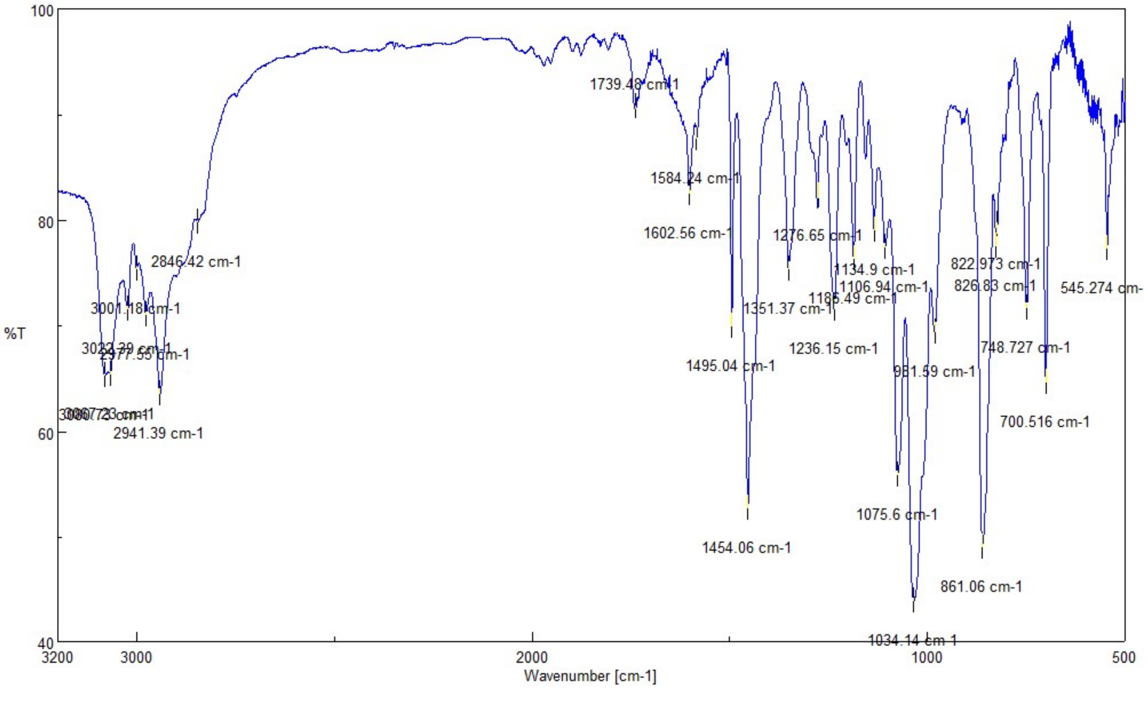

Fig. S23 IR spectrum of **7a** (KBr, solid, room temperature).

Synthesis of 7b

i) One equivalent of 4-thf

To a green solution of **4-thf** (381 mg, 479 μmol) in THF (10 mL) was added 5-phenyl-2-pentyne (65.0 mg, 499 μmol) dropwise at 0 °C. The reaction mixture was warmed up to room temperature and stirred additionally for 1 hour, resulting in a gradual color change to clear green and white solid The white solid was removed by centrifugation and the green supernatant was collected. All volatile materials of the obtained green solution was removed under vacuum. The greenish residue was suspended in DME (10 mL). The green suspension was transferred to another Schlenk tube and layered with hexane (15 mL) at –20 °C. The supernatant was removed by cannula-transfer and the green solid was dried under vacuum to yield **7b** (67.1 mg, 125 μmol, 26% yield based on **4-thf**).

ii) Two equivalents of 4-thf

To a green solution of **4-thf** (811 mg, 1.02 mmol) in THF (10 mL) was added 5-phenyl-2-pentyne (72.0 mg, 499 μmol) dropwise at 0 °C. The reaction mixture was warmed up to room temperature and stirred additionally for 1 hour, resulting in a gradual color change to light green along with formation of a white solid. The white solid was removed by centrifugation and the green supernatant was collected. All volatile materials of the obtained green solution was removed under vacuum. The green residue was suspended in DME (30 mL). After filtration, the green filtrate was transferred to another Schlenk tube and concentrated to ca. 10 mL, and layered with hexane (10 mL) at –20 °C. The green solid was collected and dried under vacuum to yield **7b** (256 mg, 476 mmol, 95% yield based on 5-phenyl-2-pentyne).

7b: IR (cm^{-1}; KBr): 699, 742, 799, 862, 1012, 1038, 1076, 1103, 1183, 1262, 1369, 1399, 1445, 1454, 1808, 2841, 2897, 2939, 2962, 2985. μ_{eff} (Evans' Method, DME, 20 °C): 3.98 μ_B. Anal. Calcd. for $C_{16}H_{22}O_2Br_3Cr$: C, 35.72; H, 4.12. Found: C, 34.99; H, 3.71. Multiple attempts to obtain satisfactory elemental analysis data failed.

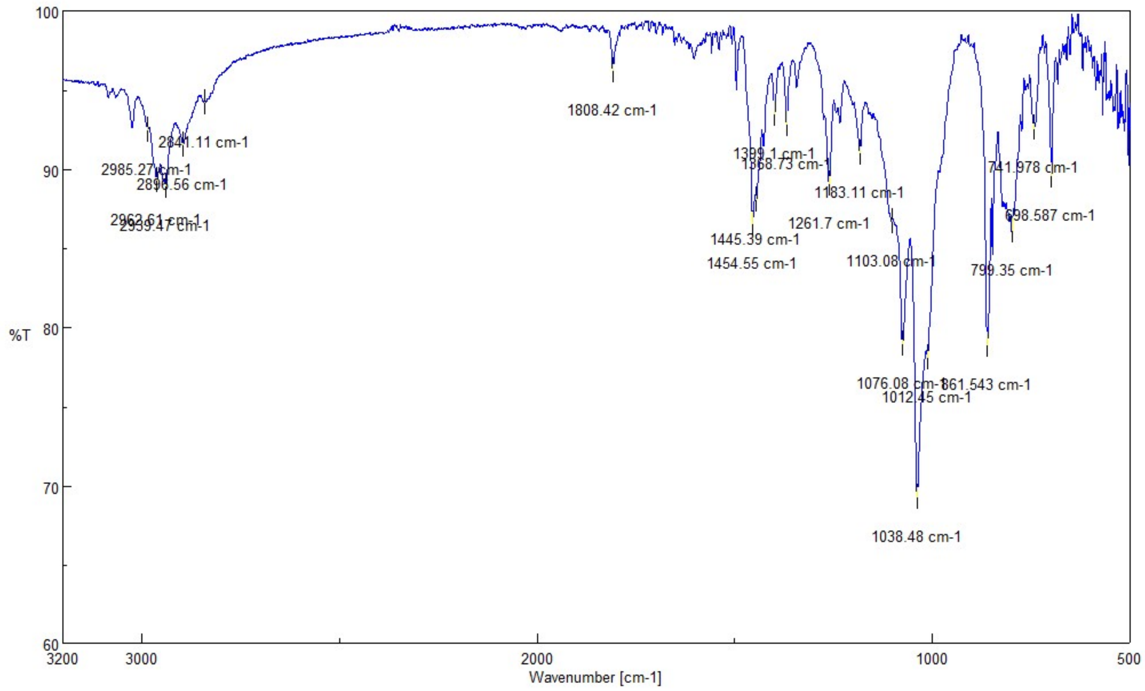

Fig. S24 IR spectrum of **7b** (KBr, solid, room temperature).

Synthesis of 7c

i) One equivalent of 4-thf

To a green solution of **4-thf** (342 mg, 430 μmol) in THF (10 mL) was added 3-hexyne (45.5 mg, 554 μmol) dropwise at 0 °C. The reaction mixture was warmed up to room temperature and stirred additionally for 1 hour, resulting in a gradual color change to light green along with formation of a white solid. The white solid was removed by centrifugation and the green supernatant was collected. All volatile materials of the obtained green solution was removed under vacuum. The green residue was dissolved in DME (5 mL). After filtration, the green filtrate was layered with hexane (5 mL) at –20 °C, resulting in formation of green crystals. The green crystals were dried under vacuum to yield **7c** (12.0 mg, 25.2 μmol, 6% yield).

ii) Two equivalents of 4-thf

To a green solution of **4-thf** (309 mg, 388 μmol) in THF (10 mL) was added 3-hexyne (16.0 mg, 195 μmol) dropwise at 0 °C. The reaction mixture was warmed up to room temperature and stirred additionally for 1 hour, resulting in a gradual color

change to light green along with formation of a white solid. The white solid was removed by centrifugation and the green supernatant was collected. All volatile materials of the obtained green solution was removed under vacuum. The green residue was dissolved in DME (5 mL). After filtration, the green filtrate was layered with hexane (5 mL) at –20 °C, resulting in formation of green crystals. The green crystals were dried under vacuum to yield **7c** (32.3 mg, 67.9 μmol, 35% yield).

7c: IR (cm^{-1}; KBr): 824, 864, 1037, 1082, 1188, 1243, 1284, 1362, 1382, 1410, 1457, 1795, 2881, 2939, 2975. μ_{eff} (Evans' Method, DME, 20 °C): 3.69 μ_B. Anal. Calcd. for $C_{11}H_{20}O_2Br_3Cr$: C, 27.76; H, 4.25. Found: C, 27.48; H, 4.15.

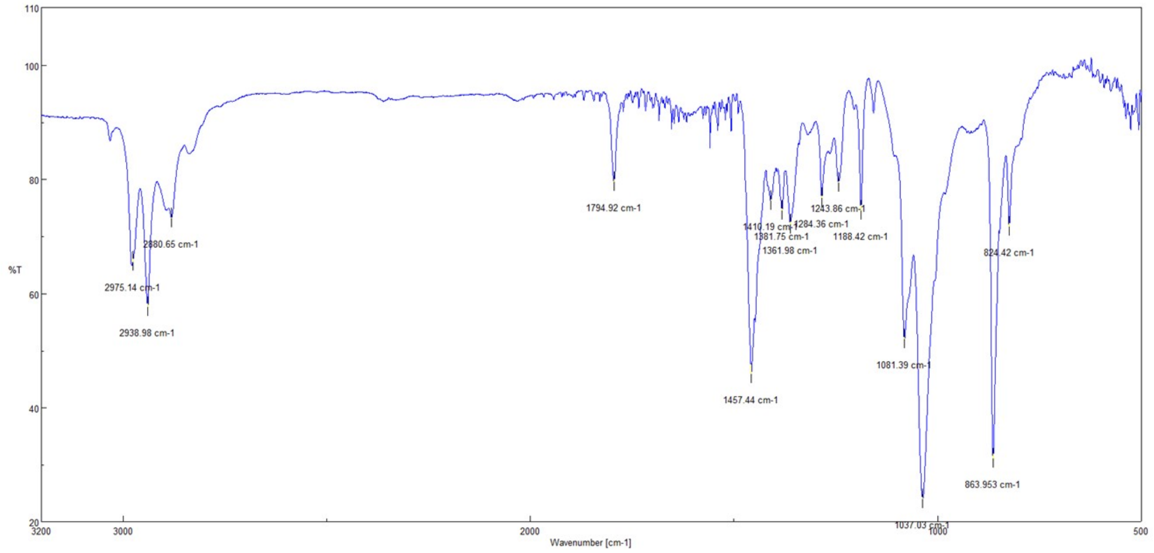

Fig. S25 IR spectrum of **7c** (KBr, solid, room temperature).

Synthesis of 7a-^{13}C

To a green solution of **4-^{13}C** (173 mg, 217 μmol) in THF (10 mL) was added 4-phenyl-1-butyne (13.2 mg, 100 μmol) dropwise at 0 °C. The reaction mixture was warmed up to room temperature and stirred additionally for 1 hour. Insoluble materials were removed by centrifugation and the green supernatant was collected. All volatile materials of the obtained green solution was removed under vacuum. The green residue was dissolved in DME (5 mL). After filtration, the green filtrate was layered with hexane (5 mL) at –20 °C. The green solid was dried under vacuum to yield **7a-^{13}C** (12.5 mg, 23.8 μmol, 24% yield).

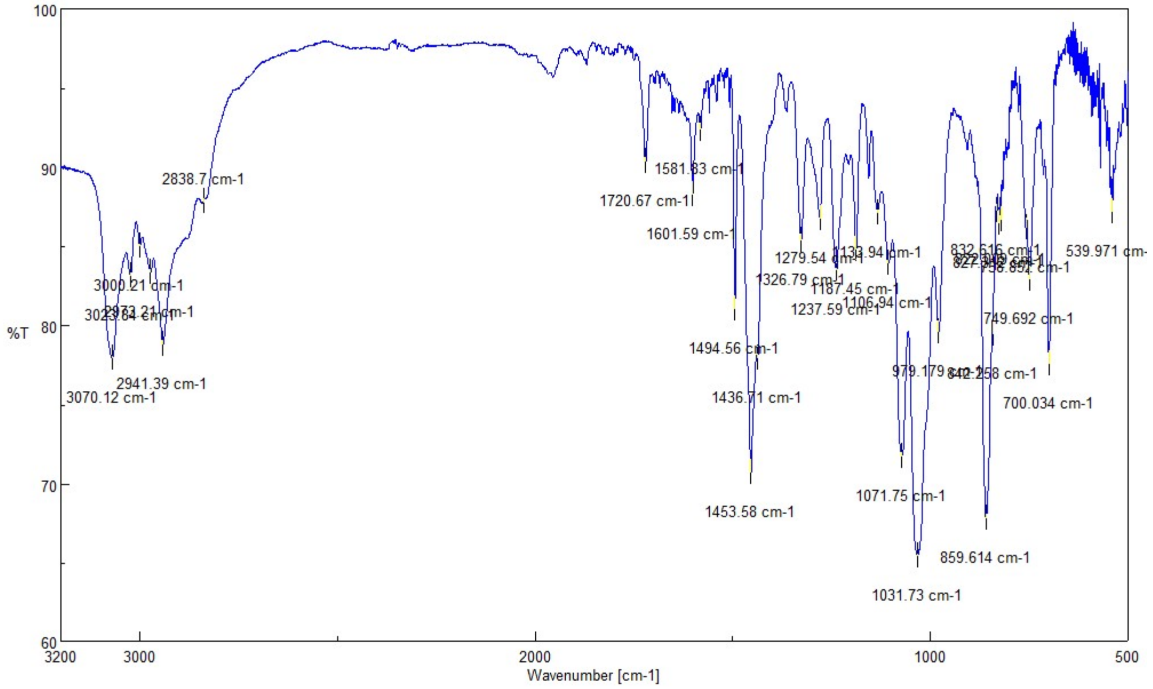

Fig. S26 IR spectrum of **7a-^{13}C** (KBr, solid, room temperature).

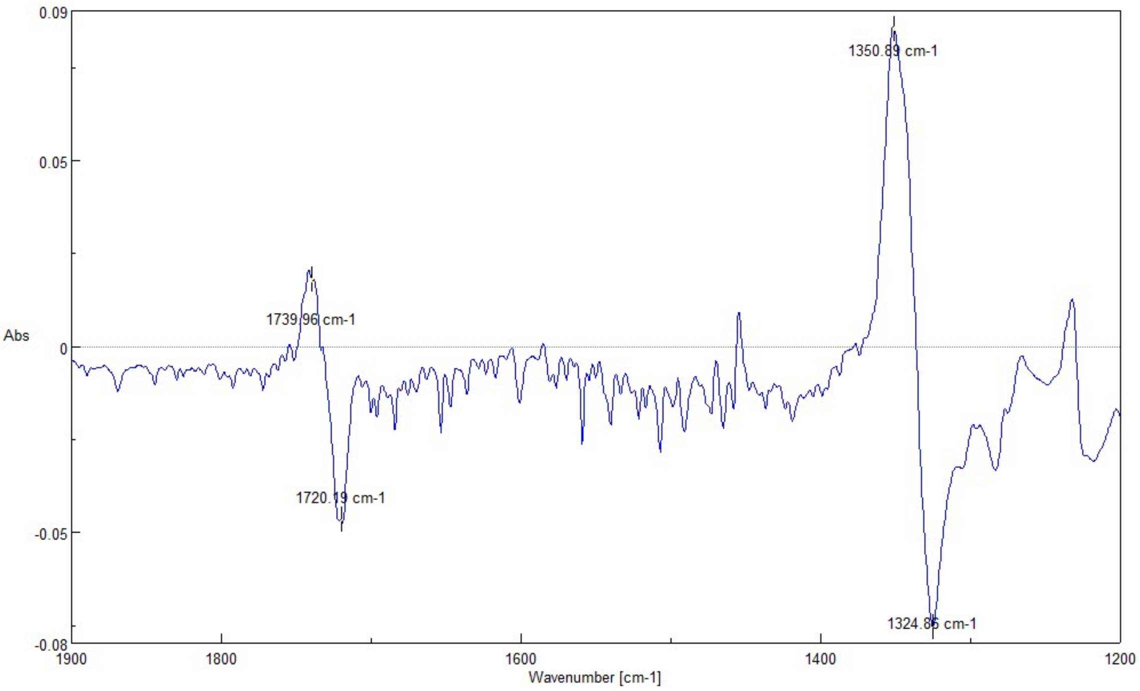

Fig. S27 Difference IR spectrum of **7a**/**7a-^{13}C** (positive: **7a**, negative: **7a-^{13}C**).

Synthesis of 7b-^{13}C

To a green solution of **4-^{13}C** (168 mg, 211 μmol) in THF (10 mL) was added 5-phenyl-2-pentyne (14.8 mg, 103 μmol) dropwise at 0 °C. The reaction mixture was warmed up to room temperature and stirred additionally for 1 hour. Insoluble materials were removed by centrifugation and the green supernatant was collected. All volatile materials of the obtained green solution was removed under vacuum. The green residue was suspended in DME (20 mL). After filtration, the green filtration was layered with hexane (15 mL) at –20 °C, resulting in formation of a green solid. The green solid was dried under vacuum to yield **7b-^{13}C** (23.2 mg, 43.1 μmol, 42% yield).

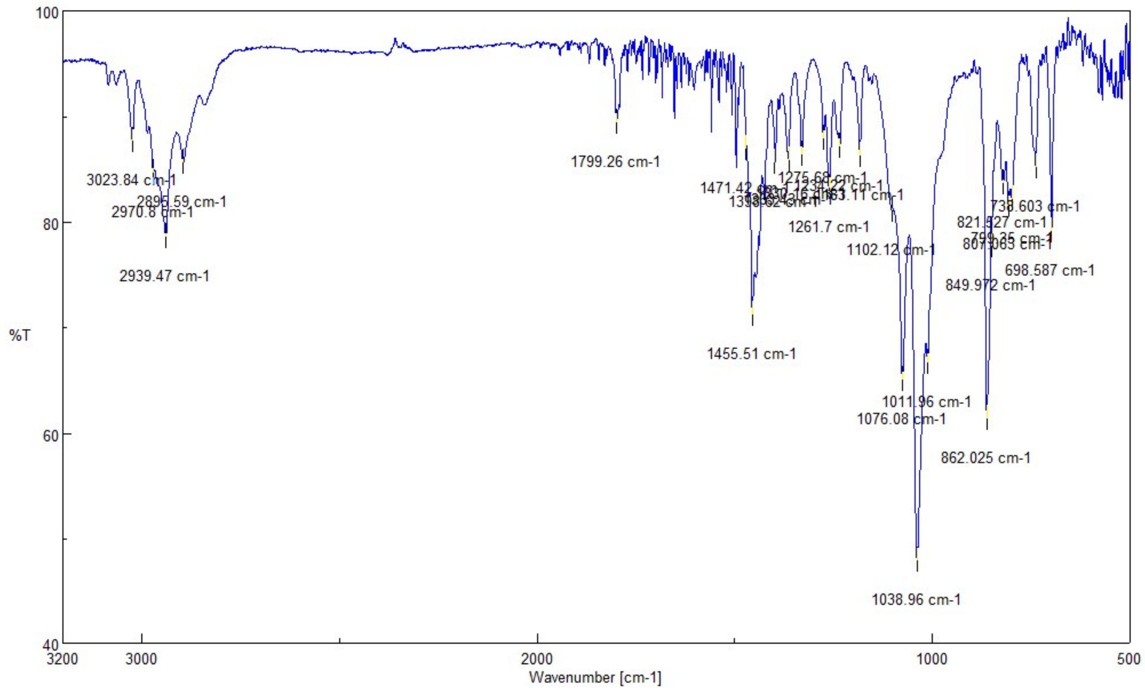

Fig. S28 IR spectrum of **7b-^{13}C** (KBr, solid, room temperature).

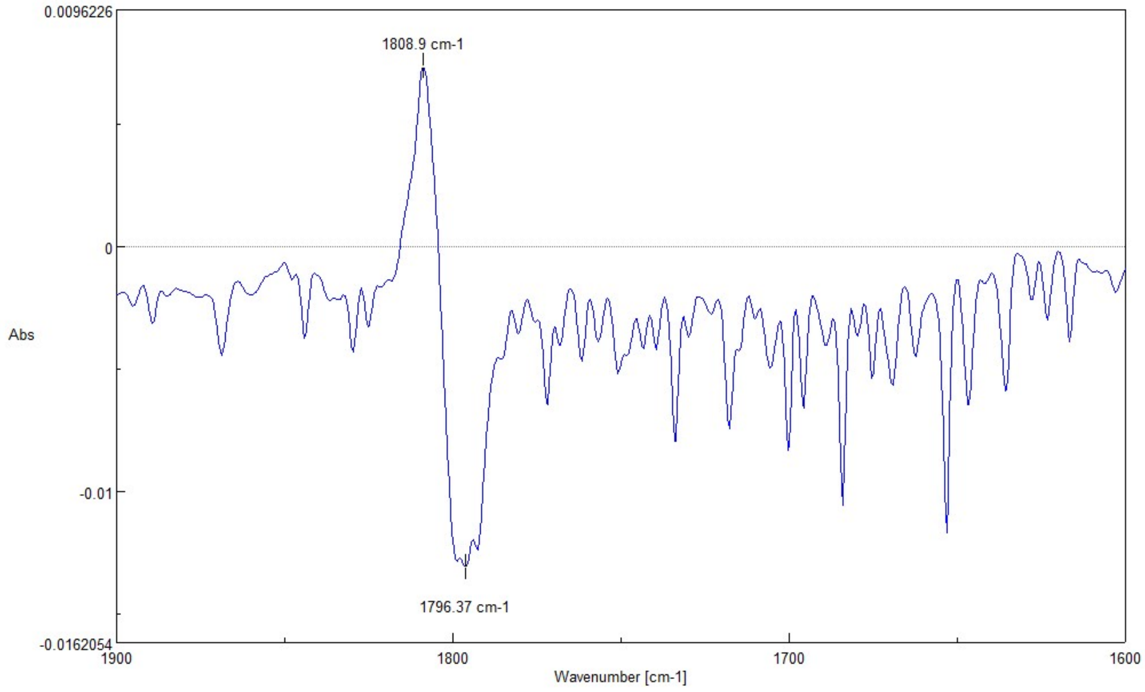

Fig. S29 Difference IR spectrum of **7b**/**7b-^{13}C** (positive: **7b**, negative: **7b-^{13}C**).

Synthesis of 7c-^{13}C

To a green solution of **4-^{13}C** (244 mg, 306 μmol) in THF (10 mL) was added 3-hexyne (12.3 mg, 150 μmol) dropwise at 0 °C. The reaction mixture was warmed up to room temperature and stirred additionally for 1 hour. Insoluble materials were removed by centrifugation and the green supernatant was collected. All volatile materials of the obtained green solution was removed under vacuum. The green reside was dissolved in DME (5 mL). After filtration, the green filtrate was layered with hexane (5 mL) at –20 °C, resulting in formation of green crystals. The green crystals were dried under vacuum to yield **7c-^{13}C** (26.1 mg, 54.7 mmol, 36% yield).

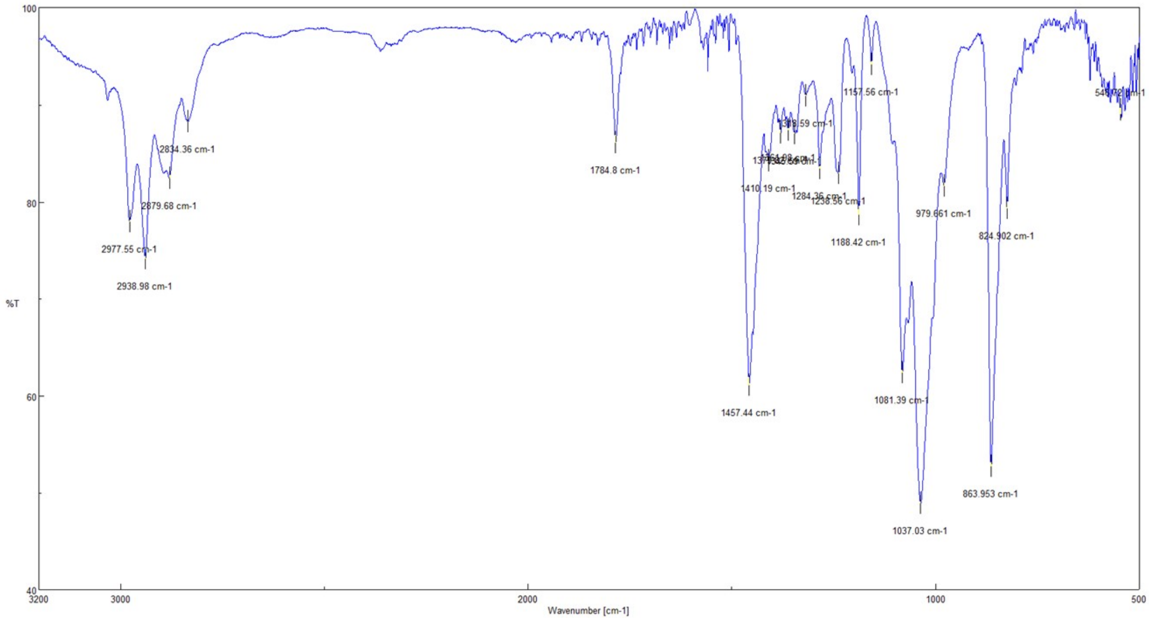

Fig. S30 IR spectrum of **7c-^{13}C** (KBr, solid, room temperature).

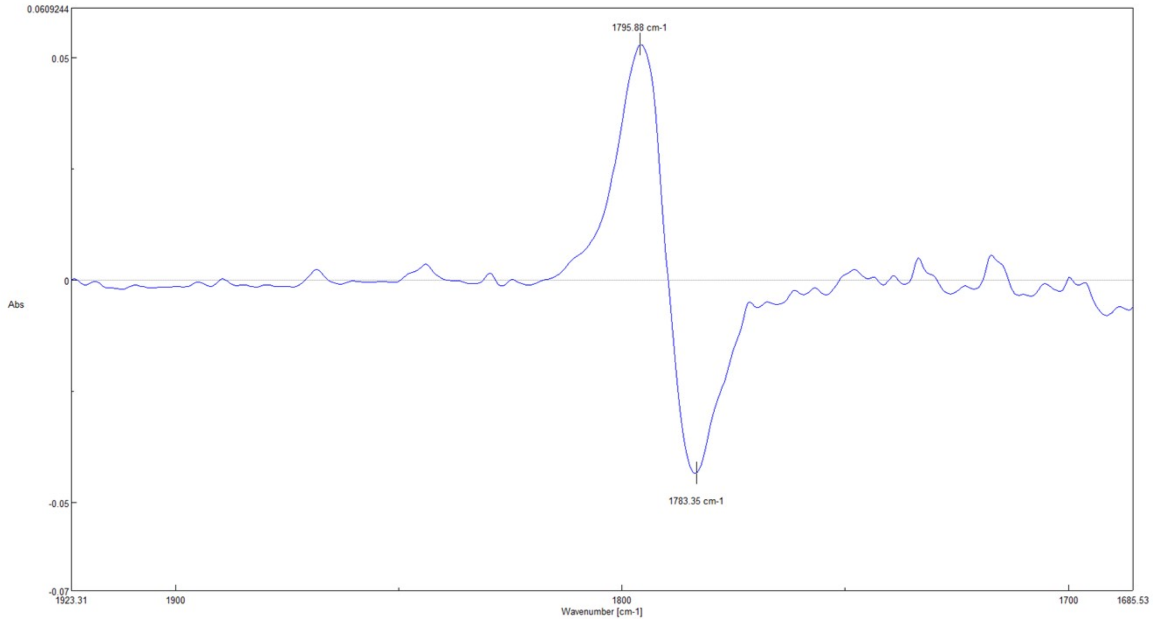

Fig. S31 Difference IR spectrum of **7c**/**7c-^{13}C** (positive: **7c**, negative: **7c-^{13}C**).

FID-GC and GC-MS Analyses

For FID-GC analyses, the solutions were transferred into a GC vial and injected into a Shimadzu GC-17A system. Separation was performed with nitrogen (3.0 mL/min, 55 cm/sec) as the carrier gas through a Zebron ZB-5MSplus GC column (30 m x 0.25 mm x 0.25 μm) with holding at 50 °C for 5 minutes, heating up to 300 °C with 10 °C/min rate, and then holding at 300 °C for 20 minutes.

For GC-MS analyses, the solutions were transferred into a GC vial and injected into a Shimadzu GC-2010 system equipped with a Shimadzu GCMS-QP2010 detector. Separation was performed with helium (2.0 mL/min, 52 cm/sec) as the carrier gas through a Zebron ZB-5MSplus GC column (30 m x 0.25 mm x 0.25 μm) with holding at 50 °C for 8 minutes, heating up to 300 °C with 10 °C/min rate, and then holding at 300 °C for 12 minutes.

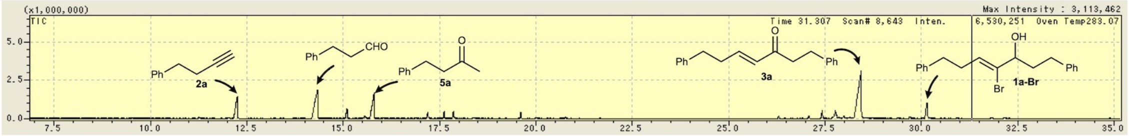

1a-Br

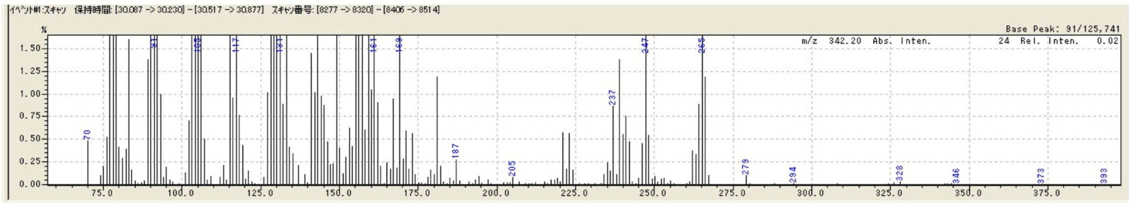

2a

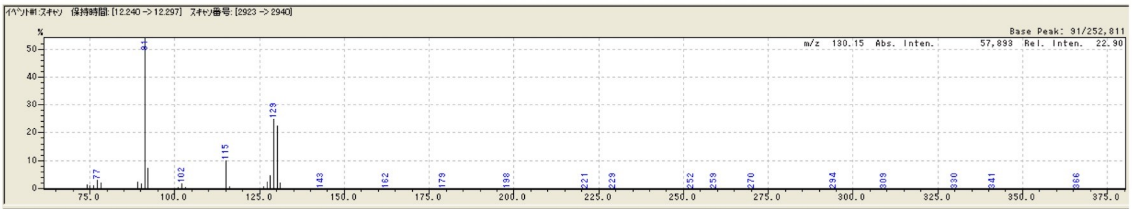

3a

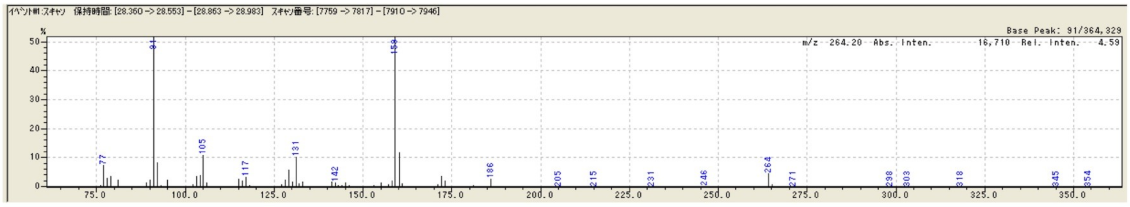

5a

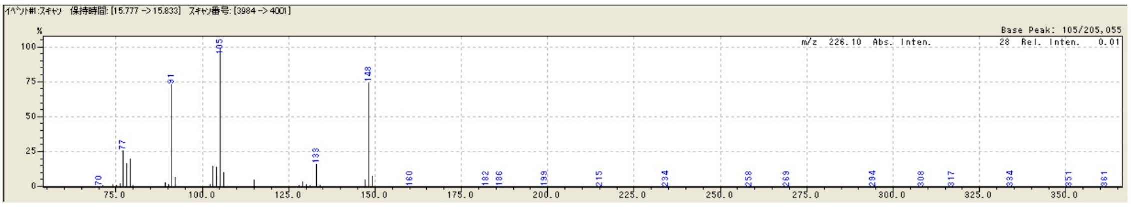

Fig. S32 GC-MS spectrum of reaction of 3-phenylpropanal with pre-mixed CBr_4 and $CrBr_2$ in THF (Top: Gas Chromatogram, Middle to Bottom: Mass spectra of **1a-Br**, **2a**, **3a**, and **5a**).

3a

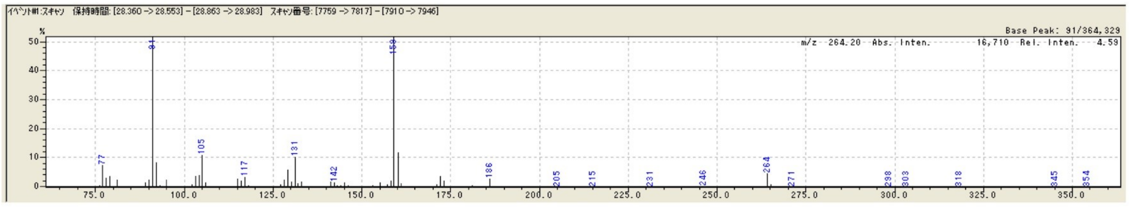

3a-^{13}C

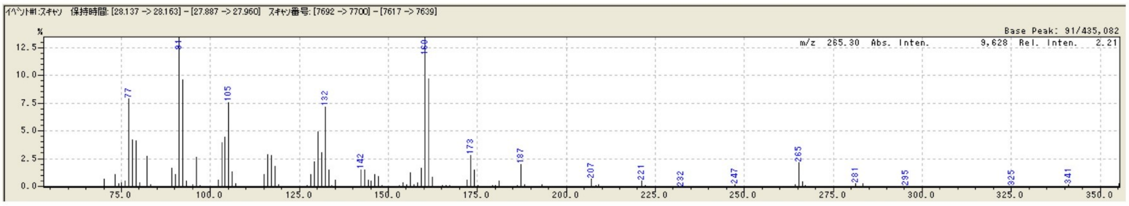

3a-*d*

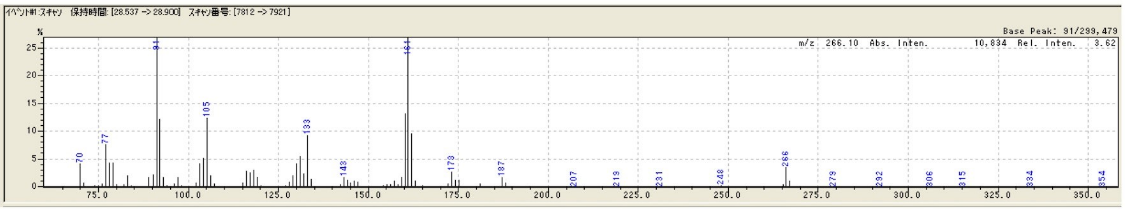

Fig. S33 Mass Spectra of **3a** (Top), **3a-^{13}C** (Middle), and **3a-*d*** (Bottom) obtained from reactions of **4-thf** with 3-phenylpropanal, **4-^{13}C** with 3-phenylpropanal, and **4-thf** with 3-phenylpropanal-*d*, respectively.

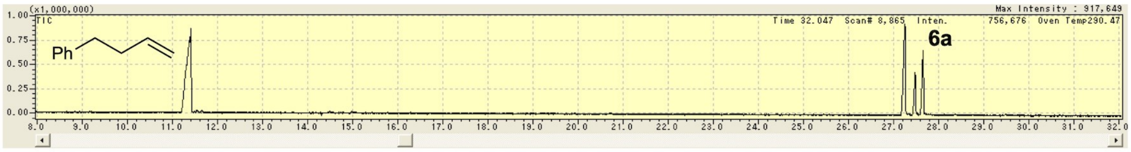

6a

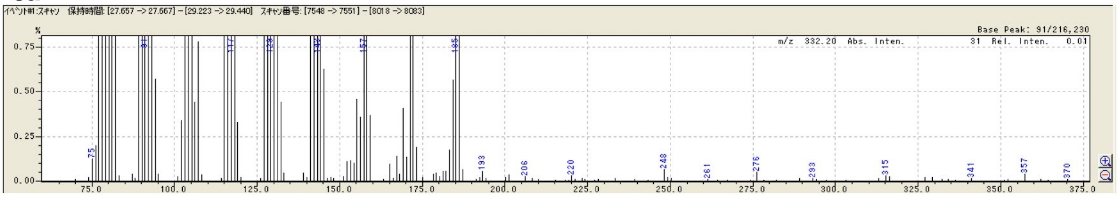

6a-^{13}C

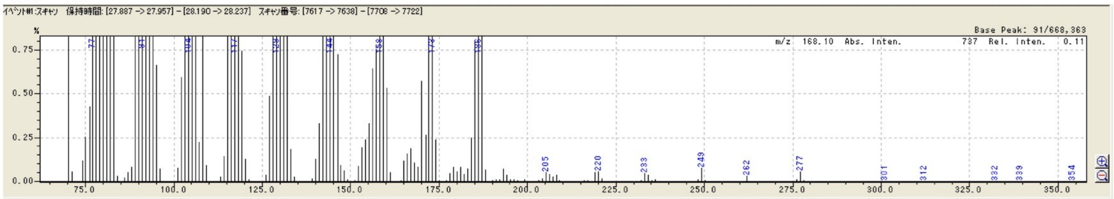

Fig. S34 GC-MS spectrum of reaction of 4-phenyl-1-butene with **4-thf** in THF (Top: Gas Chromatogram, Middle: Mass spectrum of **6a**, Bottom: Mass spectrum of **6a-^{13}C**).

X-ray Crystallography

Crystallographic data of **4-py** and **7c** are summarized in Table S1-S2 and preliminary data of **4-thf** are shown in Fig. S35. Suitable crystals for X-ray analysis were placed on the end of a micro-mount coated with NVH oil. The X-ray intensity data collection was carried out on a Rigaku Varimax with a Saturn 944+ CCD area detector using graphite-monochromated Mo-Kα radiation (λ = 0.71075 Å) at 100(2) K. Preliminary indexing was performed from a set of twelve frames. Equivalent reflections were merged, and the collected images were processed by a Rigaku CrystalClear program. The initial structures were determined by the direct or Patterson method on SHELXS.[9] The further structure determination was performed by Fourier transform method and refined by least squares method on SHELXL.[10,11] All reflections were used during refinement with the exception of affected reflections by the beam-stopper. Non-hydrogen atoms were refined anisotropically and hydrogen atoms were refined using riding models. The thermal ellipsoids of the disorders were fixed by SHELXL restraints. For single crystals of **4-thf**, the poor quality and twin property of the obtained crystals resulted in only preliminary data by X-ray diffraction (Fig. S35). The conclusive data of the solid-state structure were obtained by the pyridine adduct **4-py**, which was prepared quantitatively by addition of pyridine to a THF solution of **4-thf**. The suitable crystal of **4-py** was grown from a saturated THF solution of **4-py** at –20 °C. For **4-py**, the pyridine ligands on the chromium center and the solvated THF molecules were disordered over two positions. These results were checked by the IUCR's CheckCIF routine. The alerts in the output are related to the disordered groups. The residual Q-peaks (>1) still remain in some data, but those Q-peaks are on unrealistic positions to locate any atom.

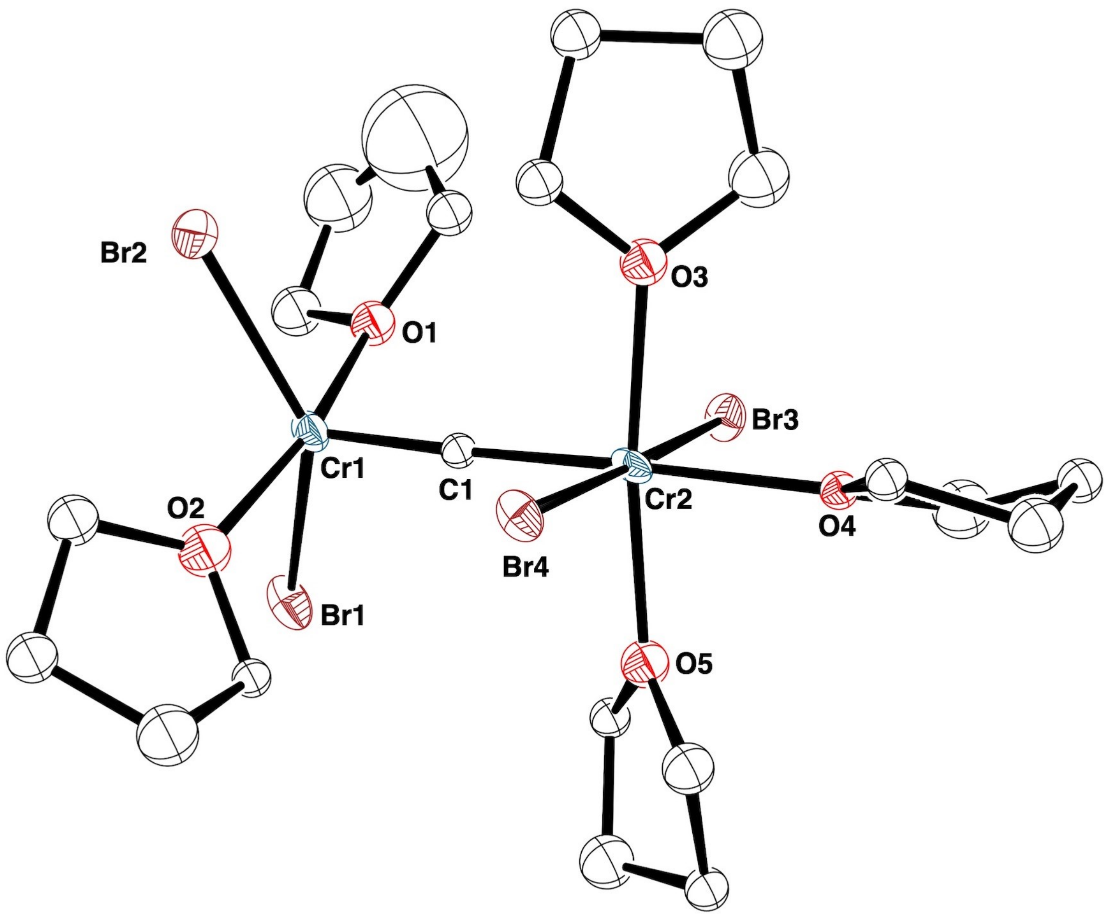

Fig. S35 Preliminary structure of **4-thf** with ellipsoids at 50% probability level. Hydrogen atoms have been omitted for clarity. The oxygen and carbon atoms have been refined and displayed isotropically. *Orthorhombic* $P2_12_12_1$, a = 12.081(14) Å, b = 12.649(15) Å, c = 19.58(2) Å, Z = 4. Bond distances Cr1–C1: 1.74(3) Å, Cr2–C1: 1.96(3) Å, Cr1–Br1: 2.506(7) Å, Cr1–Br2: 2.509(7) Å, Cr2–Br3: 2.531(6) A, Cr2–Br4: 2.534(6) Å. Bond angles Cr1–C1–Cr2: 176.8(18)°; C1–Cr1–Br1: 106.6(10)°, C1–Cr1–Br2: 111.2(10)°, C1–Cr2–Br3: 89.0(9)°, C1–Cr2–Br4: 89.5(9)°.

Table S1. Summary of Structure Determination of 4-py·THF

Empirical formula	$C_{30}H_{33}O_1N_5Br_4Cr_2$
Formula weight	903.25
Temperature	100(2) K
Wavelength	0.71075 Å
Crystal system	*Monoclinic*
Space group	$P2_1/n$ (No. 14)
Cell constants:	
a	9.184(3) Å
b	22.258(6) Å
c	17.025(5) Å
α	90°
β	103.307(5)°
γ	90°
Volume	3386.8(17) $Å^3$
Z	4
Density (calculated)	1.771 Mg/m^3
Absorption coefficient	5.392 mm^{-1}
F(000)	1776
Crystal size	0.08 x 0.04 x 0.01 mm^3
Theta range for data collection	2.456 to 27.473°
Index ranges	$-11 \le h \le 11, -28 \le k \le 16, -21 \le l \le 21$
Reflections collected	18741
Independent reflections	7465 [R (int) = 0.0943]
Completeness to theta = 27.47°	96.3 %
Absorption correction	Semi-empirical from equivalents
Max. and min. transmission	1.000 and 0.580
Refinement method	Full-matrix least-squares on F^2
Data / restraints / parameters	7465 / 207 / 471
Goodness-of-fit on F^2	1.018
Final R indices [I>2sigma(I)]	$R_1 = 0.0991$, $wR_2 = 0.2612$
R indices (all data)	$R_1 = 0.1324$, $wR_2 = 0.2798$
Largest diff. peak and hole	1.923 and –1.149 e.$Å^{-3}$

Table S2. Summary of Structure Determination of 7c

Empirical formula	$C_{11}H_{20}O_2Br_3Cr_1$
Formula weight	476.00
Temperature	100(2) K
Wavelength	0.71075 Å
Crystal system	*Monoclinic*
Space group	$P2_1/c$ (No. 14)
Cell constants:	
a	10.606(3) Å
b	11.192(3) Å
c	13.906(4) Å
α	90°
β	102.494(3)°
γ	90°
Volume	1611.6(8) $Å^3$
Z	4
Density (calculated)	1.962 Mg/m^3
Absorption coefficient	8.137 mm^{-1}
F(000)	924
Crystal size	0.14 x 0.06 x 0.03 mm^3
Theta range for data collection	3.936 to 27.501°
Index ranges	$-8 \le h \le 13$, $-13 \le k \le 14$, $-18 \le l \le 17$
Reflections collected	9683
Independent reflections	3663 [R (int) = 0.0309]
Completeness to theta = 27.49°	99.1 %
Absorption correction	Semi-empirical from equivalents
Max. and min. transmission	1.000 and 0.521
Refinement method	Full-matrix least-squares on F^2
Data / restraints / parameters	3663 / 0 / 158
Goodness-of-fit on F^2	1.181
Final R indices [I>2sigma(I)]	$R_1 = 0.0266$, $wR_2 = 0.0563$
R indices (all data)	$R_1 = 0.0340$, $wR_2 = 0.0584$
Largest diff. peak and hole	0.793 and –0.888 e.$Å^{-3}$

Computational Details

Density functional calculations and natural bond order analyses were carried out using the Gaussian09 program.[12] The model structures of **4-thf** and **4-py** were optimized from the C_1 symmetric structures based on the experimental solid-state geometry. The optimized structures were considered true minima if no imaginary vibration mode was obtained. DFT methods were performed at the unrestricted BLYP level using 6-31G(d,p) basis set for H, C, N, and O and LanL2TZ basis set for Br and Cr. The calculations at the unrestricted B3LYP and M06 level with LanL2TZ as well as SVP or ccpV-TZ for Cr were also attempted, but the optimized structure of **4-py** with BLYP/LanL2TZ revealed a better reproducibility compared to the solid-state structure of **4-py**. NBO analyses were performed by additional single-point calculations on each optimized geometry.

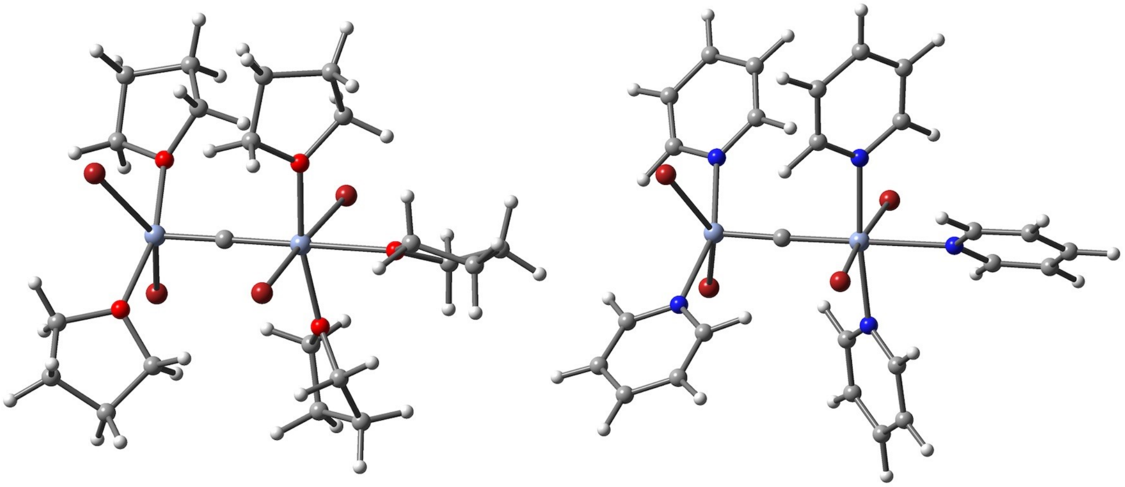

Fig. S36 Optimized structures of **4-thf** (left) and **4-py** (right) with UBLYP/LanL2TZ (Br, Cr) and 6-31g(d,p) (H, C, O, N).

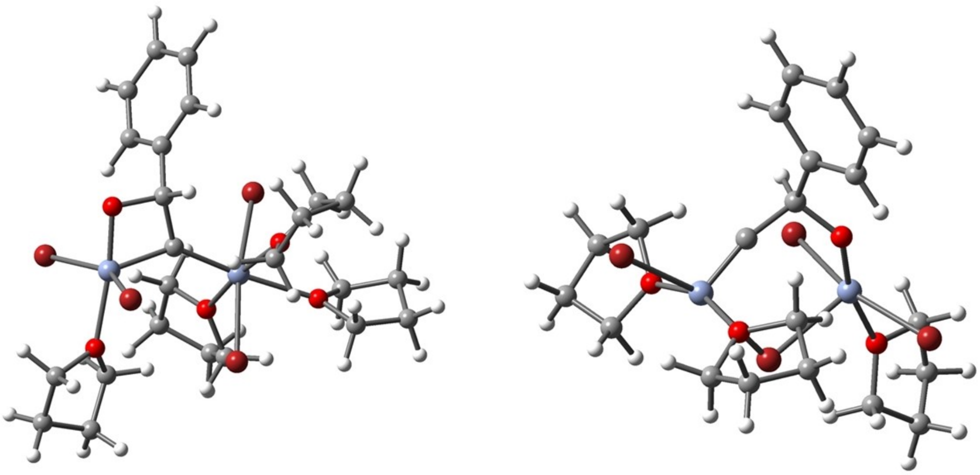

Fig. S37 Optimized structures of the intermediates formed by [2+2]-cycloaddition of the C=O bond in PhCHO to the Cr≡C bond (right) and insertion of PhCHO to the Cr–C bond (left) with UBLYP/LanL2TZ (Br, Cr) and 6-31g(d,p) (H, C, O).

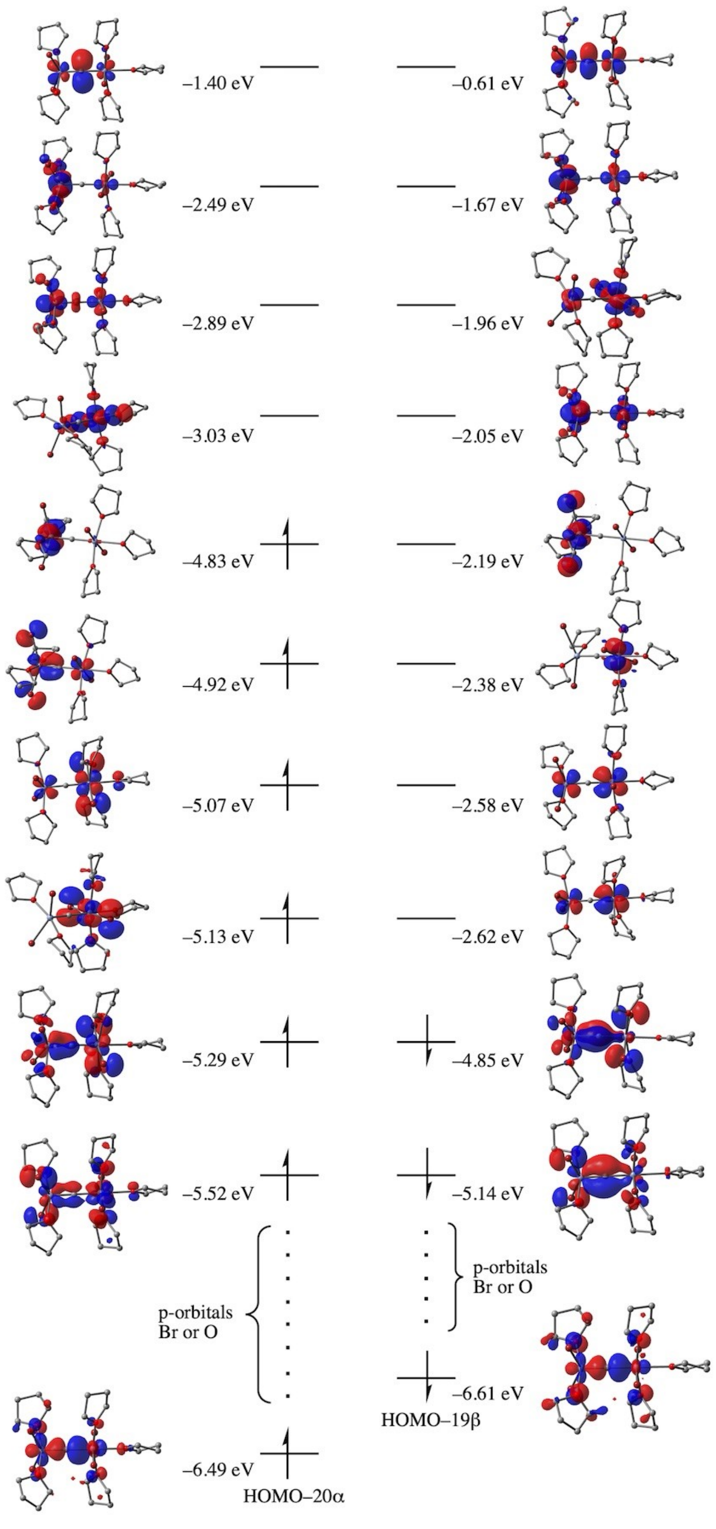

Fig. S38 Molecular orbitals of **4-thf** (isovalue = 0.04) optimized with UBLYP/LanL2TZ (Br, Cr) and 6-31g(d,p) (H, C, O).

Table S3. Cartesian Coordinates of the Optimized Geometries

The cartesian coordinates of optimized geometries are given below in the standard XYZ format (units are in Å).

==============================

4-thf

==============================

Cr	-1.438900	0.015300	-0.003000
Cr	2.164400	-0.024700	0.013100
Br	-1.522000	1.146500	2.386800
Br	-1.515600	-1.104100	-2.396500
Br	2.871200	-1.395200	2.100600
Br	2.921100	1.339500	-2.066100
C	0.452400	0.001100	0.008600
O	-3.779800	0.029300	-0.016800
C	-6.047200	-0.150300	-0.779000
H	-6.199400	-1.234900	-0.905400
H	-6.817500	0.375500	-1.362400
C	-6.053800	0.226100	0.722500
H	-6.200400	1.311700	0.847200
H	-6.833400	-0.294700	1.297900
C	-4.628400	0.249300	-1.210000
H	-4.571600	1.317400	-1.479900
H	-4.205800	-0.350200	-2.025100
C	-4.641800	-0.182500	1.168200
H	-4.595000	-1.250300	1.440600
H	-4.223100	0.415900	1.986200
O	-1.681400	-1.854500	0.902600
C	-0.981800	-2.299700	2.146200
H	-1.430200	-1.733600	2.973600
H	0.075800	-2.026600	2.046400
C	-1.250300	-3.809500	2.232900
H	-1.287000	-4.152000	3.277200
H	-0.462300	-4.377900	1.713200
C	-2.602800	-3.969200	1.495700
H	-3.440000	-3.678100	2.150600
H	-2.781200	-4.994500	1.139200
C	-2.456500	-2.985600	0.330800
H	-1.878100	-3.404300	-0.505400
H	-3.394100	-2.568000	-0.053000
O	-1.647200	1.887900	-0.909000
C	-2.419500	3.027000	-0.348300
H	-1.863200	3.422600	0.513800
H	-3.378100	2.623400	-0.003200
C	-2.505200	4.028600	-1.503500
H	-3.322800	3.761400	-2.192600
H	-2.678100	5.052100	-1.139300
C	-1.129400	3.853300	-2.192500
H	-0.350000	4.396600	-1.634300
H	-1.120100	4.212600	-3.231800
C	-0.894700	2.337400	-2.120300
H	-1.325400	1.794300	-2.972300
H	0.152400	2.040400	-1.987400
O	2.508300	1.686600	1.140900
C	2.380800	3.874800	2.041500
H	2.048500	3.649600	3.067400
H	2.154600	4.929300	1.824800
C	3.882600	3.536600	1.858100
H	4.491900	3.804100	2.733600
H	4.287800	4.066800	0.982100
C	1.708800	2.929500	1.042400
H	0.678400	2.647200	1.287500
H	1.775200	3.298700	0.006200
C	3.884800	2.014700	1.615900
H	4.600400	1.700300	0.842600
H	4.039800	1.422400	2.527400
O	2.454300	-1.749000	-1.109700
C	1.626100	-2.978900	-0.958000
H	1.473400	-3.146500	0.116200
H	0.672700	-2.750100	-1.450100
C	3.795600	-2.132200	-1.603300
H	4.184600	-1.262800	-2.146300
H	4.441200	-2.364700	-0.738600
C	3.512300	-3.365500	-2.464400
H	3.115300	-3.058500	-3.444600
H	4.416700	-3.970300	-2.626000
C	2.432800	-4.110900	-1.635200
H	2.911100	-4.749100	-0.876000
H	1.789500	-4.747500	-2.259900

==============================

4-py

==============================

Cr	-2.179900	0.015200	-0.025900
Cr	1.440400	-0.046900	0.008000
Br	-2.882300	1.260800	2.137500
Br	-2.818900	-1.186200	-2.242300
Br	1.646200	-1.150100	2.407100
Br	1.507500	1.085800	-2.369100
C	-0.477300	-0.037200	0.024900
N	-2.686000	-1.788300	0.992600
C	-3.971300	-2.234000	0.901600
H	-4.631600	-1.668000	0.243400
C	-4.432100	-3.359100	1.593600
H	-5.473200	-3.667700	1.484500
C	-3.536500	-4.069200	2.411400
H	-3.865600	-4.954300	2.960500
C	-2.212100	-3.615400	2.506200
H	-1.476900	-4.129400	3.127900
C	-1.823100	-2.474700	1.789700
H	-0.807400	-2.083600	1.850800
N	-2.476900	1.877500	-1.035900
C	-1.551100	2.874100	-1.052400

H	-0.586000	2.632800	-0.615200
C	-1.810100	4.133200	-1.610500
H	-1.026400	4.892600	-1.602000
C	-3.070400	4.387900	-2.172700
H	-3.299600	5.360500	-2.613900
C	-4.029700	3.361500	-2.161000
H	-5.023200	3.504100	-2.589400
C	-3.696500	2.127100	-1.591700
H	-4.407500	1.300600	-1.585900
N	1.539500	-2.006600	-0.907900
C	0.735100	-2.311100	-1.964700
H	0.041800	-1.535200	-2.282700
C	0.774600	-3.556900	-2.605400
H	0.101300	-3.744400	-3.443200
C	1.673200	-4.534900	-2.151500
H	1.724700	-5.514000	-2.633100
C	2.500700	-4.227600	-1.059000
H	3.212800	-4.952700	-0.660700
C	2.404900	-2.962900	-0.465500
H	3.017000	-2.694600	0.393700
N	3.775800	-0.056300	-0.057800
C	4.442800	-0.381300	-1.199000
H	3.822000	-0.607300	-2.065900
C	5.841800	-0.412000	-1.283400
H	6.317900	-0.685900	-2.226700
C	6.600100	-0.082700	-0.148200
H	7.691900	-0.093200	-0.183000
C	5.922100	0.260000	1.032900
H	6.462700	0.523600	1.943800
C	4.520200	0.255300	1.038100
H	3.960200	0.490700	1.943100
N	1.582500	1.904600	0.947600
C	2.452500	2.854600	0.499800
H	3.027700	2.596800	-0.387800
C	2.596800	4.100600	1.122900
H	3.310700	4.820900	0.719400
C	1.814200	4.395500	2.251300
H	1.904600	5.359500	2.757000
C	0.908200	3.426000	2.709500
H	0.267100	3.606800	3.573700
C	0.819300	2.199200	2.038300
H	0.119500	1.429700	2.357600

==============================

[2+2]-cycloaddition

==============================

Br	2.411400	1.398700	-1.438600
Br	0.296100	-2.582900	1.088400
Cr	1.191000	-0.455500	-0.099700
Cr	-2.319500	0.553700	0.544000
C	-0.403800	0.688200	0.248300
O	-1.938900	2.196100	1.025500
C	-0.484300	2.098500	0.810800
H	-0.033800	2.112000	1.813600
C	-0.027200	3.330500	0.061800
C	0.824000	4.235500	0.707300
C	-0.496200	3.630500	-1.224500
C	1.222800	5.413000	0.072400
H	1.174800	4.019600	1.713400
C	-0.103900	4.810400	-1.855600
H	-1.166400	2.941900	-1.726000
C	0.759300	5.702300	-1.212500
H	1.886500	6.104500	0.583300
H	-0.472700	5.034500	-2.852400
H	1.063700	6.619800	-1.707900
O	3.169300	-1.641400	-0.326900
C	5.210000	-2.696200	0.387500
H	5.367300	-3.725700	0.052200
H	5.863100	-2.521900	1.246000
C	5.471300	-1.713900	-0.768400
H	5.754000	-0.725200	-0.392000
H	6.252900	-2.056800	-1.451100
C	3.726800	-2.471700	0.740700
H	3.583300	-1.925300	1.673400
H	3.156300	-3.400800	0.792700
C	4.107800	-1.644800	-1.438000
H	3.927200	-2.533100	-2.058200
H	3.923600	-0.743400	-2.018900
O	0.553000	-1.281900	-1.890500
C	-0.008500	-0.512300	-3.019400
H	0.735900	0.234200	-3.297700
H	-0.911100	-0.018900	-2.659500
C	-0.241700	-1.543700	-4.118000
H	-0.161300	-1.092600	-5.109900
H	-1.235000	-1.995300	-4.028700
C	0.849800	-2.587800	-3.839500
H	1.819800	-2.239700	-4.209400
H	0.638200	-3.562200	-4.286700
C	0.848300	-2.656800	-2.319800
H	0.056000	-3.304300	-1.936400
H	1.791000	-2.947200	-1.864100
O	2.046200	0.201800	1.681300
C	3.291400	0.981500	1.796200
H	3.083100	1.989200	1.432600
H	4.032400	0.514600	1.149100
C	3.631200	0.953500	3.281100
H	4.210700	0.058400	3.531400
H	4.213500	1.830500	3.573700
C	2.245400	0.897500	3.939200
H	1.780800	1.889300	3.941900
H	2.270600	0.528500	4.967300
C	1.488600	-0.053800	3.023900
H	1.678900	-1.102500	3.260900
H	0.412000	0.106300	2.975600
Br	-3.353700	1.179600	-1.710900
Br	-2.532400	-0.129100	2.996300
O	-3.078000	-1.361600	0.097100
C	-2.686700	-2.210200	-1.025100
H	-2.955500	-1.690100	-1.950300
H	-1.610000	-2.336300	-0.952000
C	-4.400800	-1.775200	0.594600
H	-4.261900	-2.067800	1.636200
H	-5.070600	-0.913300	0.543800

C	-4.830300	-2.933500	-0.306200
H	-5.413200	-3.673400	0.247300
H	-5.440700	-2.567900	-1.137600
C	-3.494100	-3.483100	-0.828100
H	-3.599600	-4.047800	-1.757800
H	-3.015200	-4.124300	-0.081400

===============================

insertion

===============================

Br	-4.342700	-0.940500	0.457100
Br	0.798000	-0.936200	1.407400
Cr	-1.821900	-0.730300	0.622400
Cr	2.036800	0.362200	-0.443200
C	-1.337000	0.180500	-0.710100
O	0.662600	1.343100	-1.145900
C	-0.563500	0.957300	-1.724400
H	-0.394500	0.284100	-2.581700
C	-1.358100	2.172800	-2.203700
C	-2.346800	2.009300	-3.182300
C	-1.122600	3.443400	-1.667200
C	-3.096700	3.103900	-3.615200
H	-2.526000	1.026400	-3.611200
C	-1.870200	4.538600	-2.106400
H	-0.342500	3.568600	-0.925000
C	-2.860600	4.371800	-3.077100
H	-3.858600	2.967700	-4.377200
H	-1.675400	5.523400	-1.691000
H	-3.440200	5.225000	-3.417600
O	-1.599200	-2.603600	-0.133300
C	-1.535600	-4.487700	-1.554800
H	-0.466200	-4.706000	-1.635600
H	-2.051100	-4.968600	-2.389300
C	-2.078700	-4.926200	-0.185200
H	-3.170500	-4.995300	-0.210100
H	-1.679200	-5.887300	0.146400
C	-1.769700	-2.982900	-1.540800
H	-2.787900	-2.713300	-1.835000
H	-1.040000	-2.408900	-2.112600
C	-1.639900	-3.789800	0.734100
H	-0.631400	-3.930100	1.132300
H	-2.338600	-3.593000	1.551300
O	-1.972100	0.752600	2.005300
C	-2.622100	0.572200	3.309600
H	-1.958300	-0.036700	3.931800
H	-3.560200	0.045400	3.131800
C	-2.785200	1.987500	3.851900
H	-3.715800	2.430100	3.482900
H	-2.805500	2.000200	4.944000
C	-1.567600	2.717100	3.262300
H	-0.665600	2.492500	3.840400
H	-1.694800	3.801700	3.231500
C	-1.463400	2.124500	1.862600
H	-2.107400	2.633100	1.140300
H	-0.446700	2.058900	1.474700
Br	2.740800	2.398300	0.999100
Br	1.896200	-1.446300	-2.260400
O	3.898300	-0.470100	0.078600
C	4.315900	-0.885100	1.423500
H	3.838500	-0.219500	2.143100
H	3.957800	-1.907900	1.568200
C	5.069500	-0.266900	-0.778200
H	4.815300	-0.655200	-1.765200
H	5.265300	0.809300	-0.836900
C	6.189900	-1.013600	-0.067100
H	6.157700	-2.078400	-0.319200
H	7.173400	-0.622500	-0.337700
C	5.840100	-0.792000	1.412600
H	6.162400	0.202900	1.735400
H	6.293300	-1.532500	2.075800

References

(1) T. Hosoya, M. Wakao, Y. Kondo, H. Doi and M. Suzuki, *Org. Biomol. Chem.*, 2004, **2**, 24-27.

(2) D. F. Evans, *J. Chem. Soc.*, 1959, 2003-2005.

(3) G. A. Bain and J. F. Berry, *J. Chem. Educ.*, 2008, **85**, 532-536.

(4) D. G. Holah and J. P. Fackler, Jr., *Inorg. Synth.*, 1967, **10**, 26-33.

(5) F. A. Cotton, S. A. Duraj, G. L. Powell and W. J. Roth, *Inorg. Chim. Acta*, 1986, **113**, 81-85.

(6) Z. Liu, S. Sang, T. G. Hartman, C.-T. Ho and R. T. Rosen, *Photochem. Anal.*, 2005, **16**, 252-256.

(7) H.-P. Guan, M. B. Ksebati, Y.-C. Cheng, J. C. Drach, E. R. Kern and J. Zemlicka, *J. Org. Chem.*, 2000, **65**, 1280-1290.

(8) B. Twamley, R. Zehnder and P. J. Shapiro, *Acta Crystallogr.*, 2001, **E57**, m80-m81.

(9) G. M. Sheldrick, *Acta Crystallogr.*, 2008, **A64**, 112-122.

(10) G. M. Sheldrick, *Acta Crystallogr.*, 2015, **C71**, 3-8.

(11) $R_1 = \Sigma||F_o|-|F_c|| / \Sigma|F_o|$, $wR_2 = [\Sigma w(F_o^2-F_c^2)^2/\Sigma w(F_o^2)^2]^{1/2}$, $GOF = [\Sigma w(F_o^2-F_c^2)^2/(n-p)]^{1/2}$;where n = the number of reflections and p = the number of parameters refined.

(12) Gaussian 09, Revision E01, M. J. Frisch, G. W. Trucks, H. B. Schlegel, G. E. Scuseria, M. A. Robb, J. R. Cheeseman, G. Scalmani, V. Barone, B. Mennucci, G. A. Petersson, H. Nakatsuji, M. Caricato, X. Li, H. P. Hratchian, A. F. Izmayloy, J. Bloino, G. Zheng, J. L. Sonnenberg, M. Hada, M. Ehara, K. Toyota, R. Fukuda, J. Hasegawa, M. Ishida, T. Nakajima, Y. Honda, O. Kitao, H. Nakai, T. Vreven, J. A. Montgomery, Jr., J. E. Peralta, F. Ogliaro, M. Bearpark, J. J. Heyd, E. Brothers, K. N. Kudin, V. N. Stagroverov, T. Keith, R. Kobayashi, J. Normand, K. Raghayachari, A. Rendell, J. C. Burant, S. S. Iyengar, J. Tomasi, M. Cossi, N. Rega, J. M. Millam, M. Klene, J. E. Knox, J. B. Cross, V. Bakken, C. Adamo, J. Jaramillo, R. Gomperts, R. E. Stratmann, O. Yazyey, A. J. Austin, R. Cammi, C. Pomelli, J. W. Ochterski, R. L. Martin, K. Morokuma, V. G. Zakrzewski, G. A. Voth, P. Salvador, J. J. Dannenberg, S. Dapprich, A. D. Daniels, O. Farkas, J. B. Foresman, J. V. Ortiz, J. Cioslowski and D. J. Fox, Gaussian, Inc, Wallingfor CT, 2013.
